# Quality appraisal of clinical guidelines for Helicobacter pylori infection and systematic analysis of the level of evidence for recommendations

**DOI:** 10.1371/journal.pone.0301006

**Published:** 2024-04-10

**Authors:** Jiayin Ou, Jiayu Li, Yang Liu, Xiaohong Su, Wanchun Li, Xiaojun Zheng, Lang Zhang, Jing Chen, Huafeng Pan

**Affiliations:** 1 Science and Technology Innovation Center, Guangzhou University of Chinese Medicine, Guangzhou, China; 2 The Second Clinical Medicine College, Guangzhou University of Chinese Medicine, Guangzhou, China; 3 The People’s Hospital of Gaozhou, Gaozhou, China; 4 School of Chinese Materia Medica, Guangzhou University of Chinese Medicine, Guangzhou, China; 5 Clinical Medical College of Acupuncture, Moxibustion, and Rehabilitation, Guangzhou University of Chinese Medicine, Guangzhou, China; 6 School of Public Health and Management, Guangzhou University of Chinese Medicine, Guangzhou, China; King Saud University Medical City, SAUDI ARABIA

## Abstract

**Objectives:**

To systematically assess the quality of clinical practice guidelines (CPGs) for Helicobacter pylori (HP) infection and identify gaps that limit their development.

**Study design and setting:**

CPGs for HP infection were systematically collected from PubMed, Embase, the Cochrane Library, the Cumulative Index to Nursing and Allied Health Literature, and six online guideline repositories. Three researchers independently used the AGREE Ⅱ tool to evaluate the methodological quality of the eligible CPGs. In addition, the reporting and recommendation qualities were appraised by using the RIGHT and AGREE-REX tools, respectively. The distribution of the level of evidence and strength of recommendation among evidence-based CPGs was determined.

**Results:**

A total of 7,019 records were identified, and 24 CPGs met the eligibility criteria. Of the eligible CPGs, 19 were evidence-based and 5 were consensus-based. The mean overall rating score of AGREE II was 50.7% (SD = 17.2%). Among six domains, the highest mean score was for scope and purpose (74.4%, SD = 17.7%) and the lowest mean score was for applicability (24.3%, SD = 8.9). Only three of 24 CPGs were high-quality. The mean overall score of recommendation quality was 35.5% (SD = 12.2%), and the mean scores in each domain of AGREE-REX and RIGHT were all ≤ 60%, with values and preferences scoring the lowest (16.6%, SD = 11.9%). A total of 505 recommendations were identified. Strong recommendations accounted for 64.1%, and only 34.3% of strong recommendations were based on high-quality evidence.

**Conclusion:**

The overall quality of CPGs for HP infection is poor, and CPG developers tend to neglect some domains, resulting in a wide variability in the quality of the CPGs. Additionally, CPGs for HP infection lack sufficient high-quality evidence, and the grading of recommendation strength should be based on the quality of evidence. The CPGs for HP infection have much room for improvement and further researches are required to minimize the evidence gap.

## 1 Introduction

Helicobacter pylori (HP) infection is a common infection globally that is an important cause of peptic ulcer disease and gastric cancer [1], and it is especially closely related to the development of gastric cancer [2]. A study published in 2018 showed that HP infection accounted for the largest proportion of attributable cancer cases worldwide [3]. Therefore, the optimization of HP eradication therapy is essential [4, 5]. However, as the most clear and controllable factor in the development of gastric cancer [6], the treatment of antimicrobial eradication of HP has gradually become a global burden due to treatment failure caused by the development of drug resistance [7]. As a result, several national and international organizations have developed and updated HP clinical practice guidelines (CPGs) to identify alternatives and improve the efficiency of diagnosis and treatment.

There are presently available both non-invasive and invasive techniques for diagnosing HP [8, 9]. The commonly employed non-invasive methods include urea breath tests and fecal antigen tests, while the invasive diagnostic option is upper gastrointestinal endoscopy [10]. Multiple treatment options are currently available for the eradication of HP infection, including triple therapy (consisting of a proton pump inhibitor (PPI) and two antibiotics such as clarithromycin, amoxicillin, or metronidazole), non-bismuth quadruple therapy (comprising of a PPI, clarithromycin, metronidazole, and amoxicillin), and bismuth quadruple therapy (involving a PPI, bismuth salt, tetracycline, and metronidazole) [11]. However, the effectiveness of triple therapy gradually diminishes as drug resistant increases [12]. Previous studies have provided a comprehensive analysis of the limitations associated with triple therapy [13–15]. To date, there remains a lack of an efficacious vaccine or prophylactic intervention for HP [16].

CPGs are statements that assist with the healthcare decision-making of physicians and patients through a systematic review of evidence and evaluation of care options [17]. CPGs are considered to be essential tools for clinicians and decision makers to enable the selection of the most effective and cost-effective treatment for their practice [18, 19]. Trustworthy CPGs should be based on a systematic review of studies, should provide ratings of evidence quality and recommendation strength, should consider patient value, and should be developed by a multidisciplinary panel of experts [17]. However, some common problems of CPGs include a lack of clear supporting evidence or a low overall level of evidence, neglect of patients’ interests and wishes, lack of editorial independence, and poor applicability [20–24]. Although there has been an systematic review on CPGs for HP infection [25], we found that it omitted important literature, including evidence-based guidelines [26–30] and consensual-based guidelines [31–35]. In addition, the Reporting Items for Practice Guidelines in Healthcare (RIGHT) and Appraisal of Guidelines Research and Evaluation-Recommendations Excellence (AGREE-REX) tools were not used for the systematic evaluation, and there was no overall comprehensive analysis of the level of evidence and strength of recommendations in the guidelines [18].

The Appraisal of Guidelines for Research and Evaluation II (AGREE II) contains 23 items covering six domains: scope and purpose, stakeholder involvement, development rigor, clarity and expression, applicability, and editorial independence [36], and is a useful and reliable tool for evaluating guidelines [37–39]. In order to improve the quality of guideline recommendations and ensure their credibility, reliability, and implementability in clinical practice, the International Guidelines Research team developed a guidelines research and evaluation system, the AGREE-REX, which complements AGREE Ⅱ [40, 41]. RIGHT has been widely implemented as a CPG reporting standard and is a useful tool for CPG makers in clinical medicine and CPG users [42, 43]. Its 22 items, including basic information, background, evidence, recommendations, review and quality assurance, funding, benefit declaration, and management, are vital elements of the reporting required in the quality guide [44].

Thus, in this study, the AGREE II, RIGHT, and AGREE-REX tools were used to systematically evaluate the quality of CPGs for HP infection, identify the distribution of the level of evidence and strength of recommendations among these CPGs, identify the potential factors leading to the low quality of CPGs, highlight potential opportunities for improvement, and provide quality references for future CPGs for HP infection development.

## 2 Materials and methods

This study was performed and reported in reference to the Preferred Reporting Items for Systematic Review and Meta-Analysis (PRISMA) statement [45], see **S1 File.**

### 2.1 Eligibility criteria

CPGs were included if they 1) focused on the diagnosis and management of HP infection; 2) were published from January 1, 2011 to October 5, 2022; and 3) were written in English. Consistent with the methods of previous studies [46, 47], both evidence-based and consensus-based CPGs were included. If the CPGs had been updated, the latest version was included. CPGs were excluded if 1) the full text was unavailable; 2) they were editorials, comments, reviews, letters, or correspondence studies; 3) they were interpretations, translations, or adaptations of a CPG; or 4) they were a duplicate of another publication.

### 2.2 Literature search

A detailed systematic search of four scientific databases: PubMed, Embase, the Cochrane Library, and the Cumulative Index to Nursing and Allied Health Literature, was conducted. In addition, information from six online guideline libraries: the National Institute for Health and Clinical Excellence (NICE), Scottish Intercollegiate Guidelines Network (SIGN), Guidelines International Networks (GIN), Agency for Healthcare Research and Quality (AHRQ), National Health and Medical Research Council (NHMRC), and World Health Organization (WHO), was retrieved. All databases were searched in combination with medical subject terms and keywords related to HP infection, and the specific search strategy is provided in **S2 File.** The search range was from January 1, 2011, to October 5, 2022.

### 2.3 Study selection and data extraction

All records were first imported to EndNote X7.7.1 (Thomson Reuters Corporation, CA, USA), then duplicates were identified and removed. One researcher (L.Z.) screened the remaining records against titles and abstracts for relevant articles. Subsequently, two researchers (L.Z. and Y.L.) independently screened full articles according to the inclusion and exclusion criteria. When disputes arose, discussion with a third researcher (J.O.) was undertaken and a consensus reached.

Two researchers (X.Z. and J.O.) independently performed the data extraction and any disagreements between the two were resolved through discussion. For each CPG that was eventually included, the accompanying documents were comprehensively searched for a more comprehensive evaluation. In order to understand the basic information and perform further subgroup analyses, the characteristics of each CPG were extracted. The extracted variables included the type of development organization (medical society, expert panel, or government organization), country (developed or developing country), version (updated or first), development method (evidence-based or consensus-based), whether a CPG quality tool was used (yes, no, or not stated), whether a CPG methodologist was involved (yes, no, or not stated), whether a grading system was used (yes, no, or not stated), whether there was a funding source (yes, no, or not stated), scope (treatment; diagnosis and treatment; or diagnosis, treatment, and prevention), and year (2016 or earlier, or 2016 or later). CPGs were classified as an ‘expert panel’ when they were not developed by specific associations or governmental organizations.

### 2.4 Quality assessment

Three tools, the AGREE II, AGREE-REX, and RIGHT, were used to systematically evaluate the quality of included CPGs for HP infection. Before applying these CPG quality tools, all researchers received systematic training, including undertaking two training exercises available on the AGREE corporate website, and read the evaluation details in the user manuals for the three tools.

#### 2.4.1 AGREE II

The methodological quality of eligible CPGs was independently assessed by three researchers (J.L., X.S., and W.L.) using the AGREE II instrument. AGREE II [37], an internationally developed, widely accepted, and transparent tool, was used for the assessment of the methodological rigor of CPGs [48]. Each CPG was evaluated in its six domains and 23 quality items, which included ‘scope and purpose’ (1~3), ‘stakeholder involvement’ (4~6), ‘rigor of development’ (7~14), ‘clarity of presentation’ (15~17), ‘applicability’ (18~21), and ‘editorial independence’ (22~23). Each item was scored on a seven-point Likert scale, ranging from one (indicating strongly disagree) to seven (indicating strongly agree). ‘Strongly disagree’ meant that the item was completely absent from the CPG, and ‘strongly agree’ meant that the quality of the item in the CPG was high. When an item was given a score of two to six, it meant that the content of the CPG did not fully meet the criteria of AGREE II. The AGREE II scores of each researcher were collated by one researcher and recorded on a Microsoft Excel spreadsheet, and any item with a score difference of more than two points in the CPG evaluation was reevaluated by the researchers until the score difference was narrowed or a consensus was reached. For each CPG, the individual domain scores were compiled and calculated as a proportion of the maximum possible score (scaled domain score) according to the formula (score obtained–minimum possible score) / (maximum score–minimum possible score) × 100% [37].

In the overall assessment, the first overall rating item was scored on a seven-point scale and then calculated as a percentage, which was the same method used to calculate domain scores in previous studies [41, 49]. For the second global evaluation item, CPGs were classified as high quality if the three domains deemed most important achieved at least 50% of the highest possible score, which was consistent with the methods used in previous studies [41, 50, 51]. The three domains were stakeholder engagement (domain 2), rigor of development (domain 3), and editorial independence (domain 6).

#### 2.4.2 AGREE-REX

The AGREE-REX tool was used to evaluate the quality of the recommendations of included CPGs. The researchers (X.S., J.O., and J.L.) formed a consensus score for nine items in each of the three domains of AGREE-REX through in-person discussion. The three domains included ‘clinical applicability’ (evidence, applicability to target users, applicability to patients and populations), ‘values and preferences’ (of target users, patients and populations, policy- and decision-makers, and guideline makers), and ‘implementability’ (purpose, local application, and adoption). Items of the AGREE-REX tool were all evaluated using a seven-point Likert scale ranging from one (strongly disagree) to seven (strongly agree). The score of the domain was obtained according to the formula (consensus score–lowest possible score) / (highest possible score–lowest possible score) × 100%.

#### 2.4.3 RIGHT

The RIGHT statement is a tool focused on assessing the quality of CPG reporting. Here, researchers (X.S., J.O., and J.L.) evaluated each selected CPG using the RIGHT scale. The RIGHT scale contains a total of seven domains and 22 items that are considered important for the quality of CPG reporting, including ‘basic information’ (Item 1~4), ‘background’ (Item 5~9), ‘evidence’ (Item 10~12), ‘recommendations’ (Item 13~15), ‘review and quality assurance’ (Item 16~17), ‘funding, declaration, and management of interests’ (Item 18~19), and ‘other information’ (Item 20~22) [44]. Three grades were used to evaluate each item; namely, ‘reported,’ ‘partially reported,’ and ‘not reported,’ corresponding to a score of 1, 0.5, and 0, respectively. RIGHT domain score = (total number of items ‘reported’ in each domain) / (total number of items in each domain) × 100%.

### 2.5 Level of evidence and strength of recommendation

By reading the full text of the included CPGs and their attachments, the grading system applied to each CPG was determined and the number of different levels of evidence and the strength of recommendations were identified.

The Grading of Recommendation Assessment, Development, and Evaluation (GRADE) system [52, 53] has been recognized as the most ideal and commonly used method for grading evidence and specifying recommendations by many societies. Therefore, to standardize statistical results, the graded evidence and recommendations were incorporated, when possible, into this classical GRADE system.

During the reassessment process, evidence and recommendations that were not clearly described in terms of level and strength were excluded. If a recommendation was supported by multiple levels of evidence, the highest level of evidence available was selected. After the CPGs were reevaluated, the distribution of the level of evidence and the strength of recommendations across the CPGs were measured.

### 2.6 Statistical analysis

The results of the assessments were entered into a Microsoft Excel spreadsheet (Microsoft, WA, USA). The standardized score for each domain and over score of each CPG were calculated, and the overall situations are expressed as mean ± standard deviation (SD). Characteristics of the CPGs are expressed as frequencies and percentages. In addition, the distribution between the level of evidence and the strength of recommendation is expressed as frequency, percentage, mean (SD), and median (Q1–Q3). CPGs were stratified by different characteristics and a subgroup analysis of AGREE II, AGREE-REX, and RIGHT results was conducted. Differences between two groups were explored by the independent-sample t test/analysis of variance/Kruskal–Wallis (H) test. Additionally, the association among the AGREE II, AGREE-REX, and RIGHT domains was examined by Spearman’s correlation. The intraclass correlation coefficients (ICCs) with 95% CI were used to test for agreement among the three researchers and assess inter-rater reliability. Generally, an ICC of < 0.40 was classified as poor, an ICC of 0.40–0.59 was classified as fair, an ICC of 0.60–0.75 was classified as good, and an ICC of > 0.75 was classified as excellent. R 3.4.3 (http://www.R-project.org; The R Foundation), EmpowerStats 4.1 (http://www.empowerstats.com; X&Y Solutions, Inc., MA, USA), and SPSS 23.0 (IBM, IL, USA) software were used to analyze all data. p < 0.05 was considered statistically significant. The GraphPad Prism 8.0 (GraphPad Software, San Diego, CA, USA) and a data visualization tool (https://www.datawrapper.de/) were used to present results in Column bar graphs or distribution maps.

### 2.7 Ethics statement

No subjects were involved in this study, so ethical approval is not required.

## 3 Results

### 3.1 Study selection

**Fig 1:**
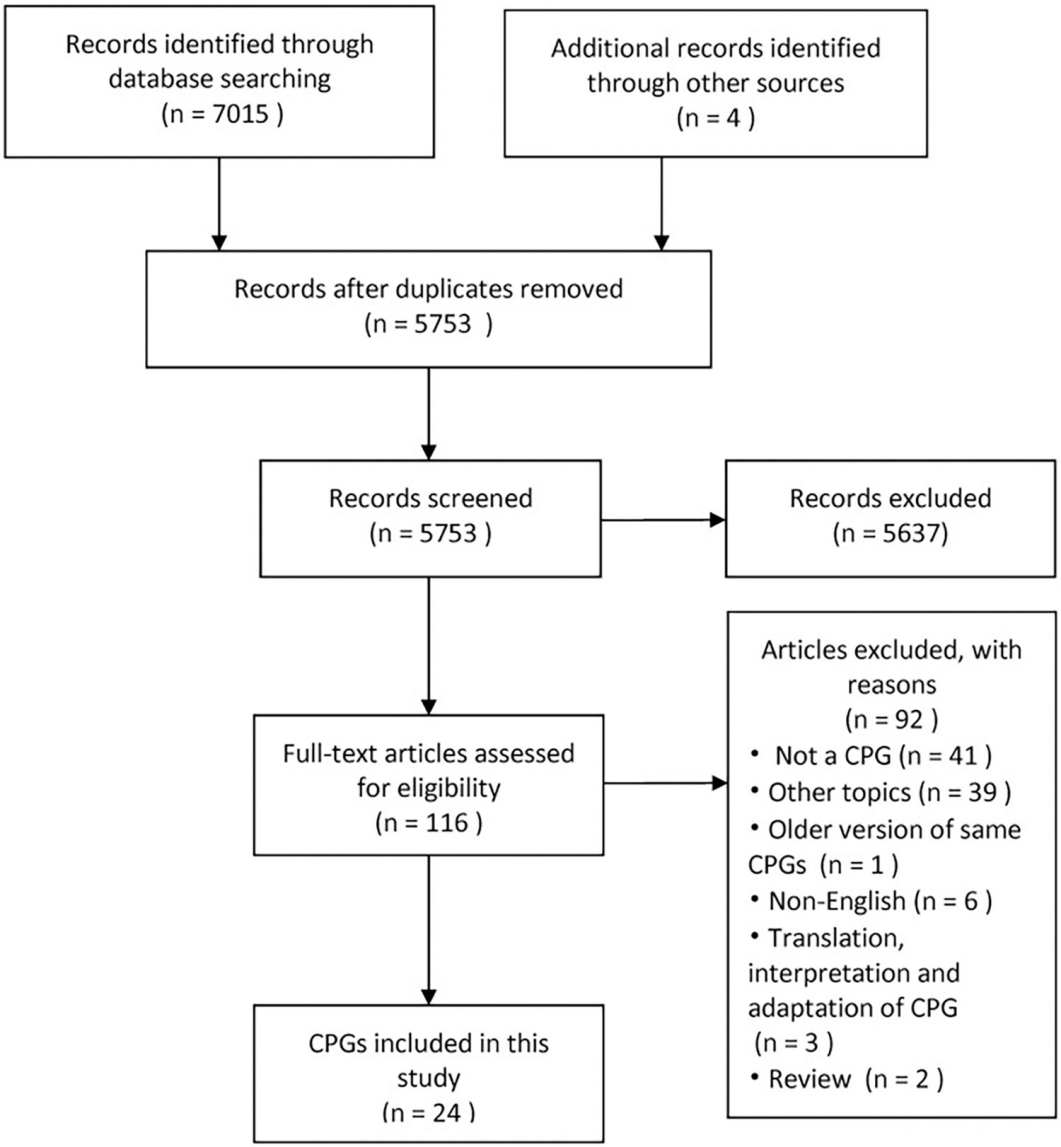
Study flow diagram.

A total of 7,015 references were obtained by searching the databases, and four more were obtained by other means. Later, 5,753 references were reviewed and deleted by EndNote, and 5,637 were removed based on the title and abstract. Overall, 116 CPGs were finally included in the full-text guideline review. Among them, 92 were removed by researchers according to the inclusion criteria, thus, 24 CPGs fully met the inclusion criteria **(Fig 1)**.

### 3.2 Characteristics of the CPGs

**Fig 2:**
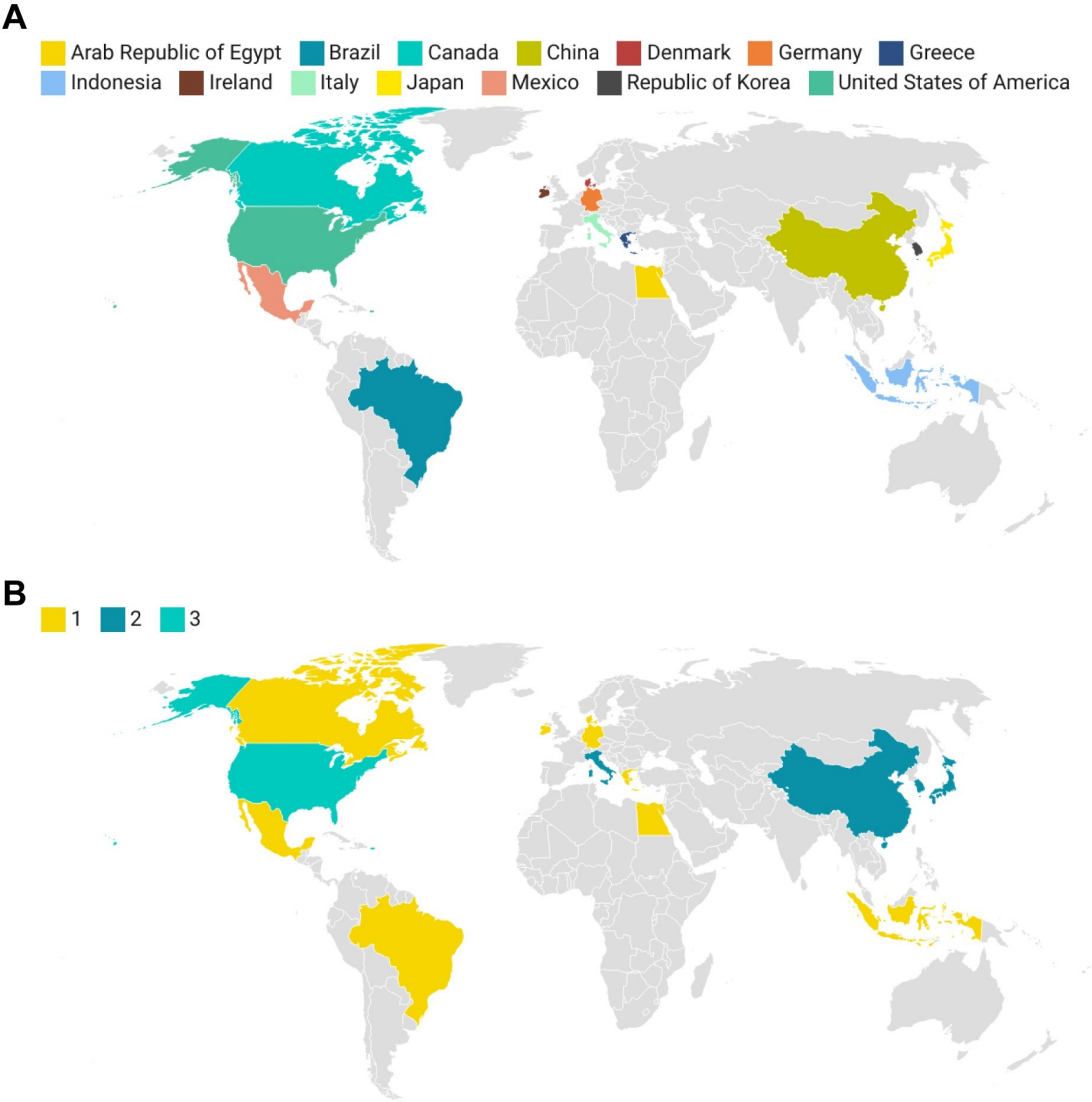
Distribution of GPGs for HP infection. (A) Geographic coverage of the CPGs for HP infection. (B) Quantity distribution of CPGs for HP infection. CPG, Clinical practice guideline; HP, Helicobacter pylori.

Among the 24 CPGs, three were developed by international collaborations; three were developed by the United States; two were developed by China, Japan, South Korea, and Italy each; and one was developed by Denmark, Mexico, Canada, Indonesia, Ireland, Egypt, Greece, Germany, Brazil, and Latin America each **(Fig 2)**. Developed countries were the main source of CPGs with 16 (66.7%), and developing countries accounted for eight (33.3%). Of these, 14 were developed by medical societies, eight by expert groups, and two by governments. Of the 24 CPGs, eight were for treatment, 13 for diagnosis and treatment, and three for diagnosis, treatment, and prevention. Most CPGs were evidence-based (n = 18), clearly stated funding sources (n = 13), and used quality tools (n = 15) **(S1 and S2 Tables)**.

### 3.3 Quality of CPG methodology

**Fig 3:**
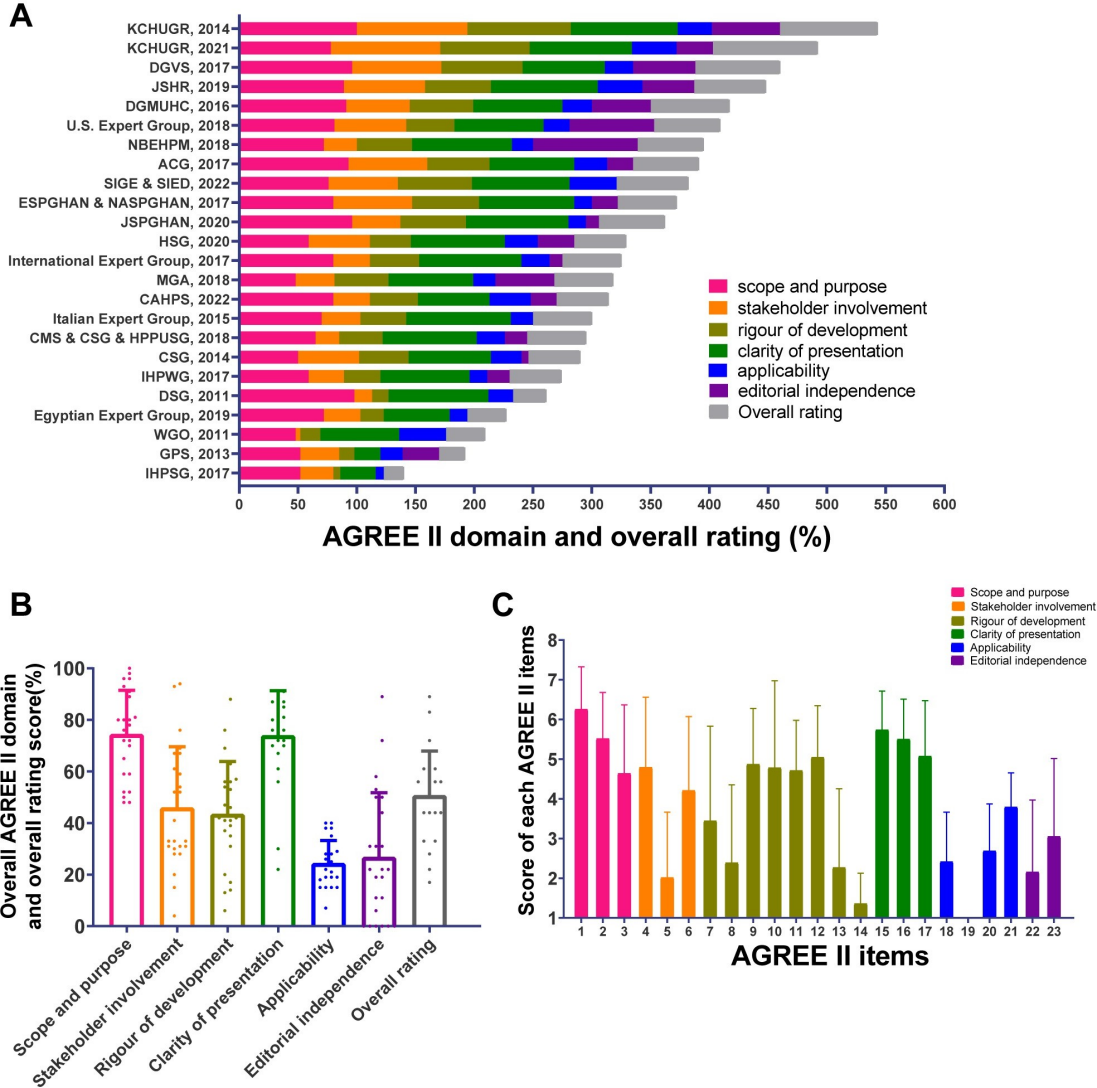
AGREE II scores. (A)AGREE II domain and overall rating in each CPGs. (B)Average score of each AGREE II domain and overall rating for all CPGs. (C) Average score of each AGREE II item for all CPGs. CPG, Clinical practice guideline; AGREE II, the Appraisal of Guidelines for Research and Evaluation II.

**Fig 3 and S4 Table** show the AGREE Ⅱ score of the included CPGs. The mean overall rating score for all CPGs was 50.7% (SD = 17.2%), and three CPGs [33, 54, 55] were high-quality. Domain 1 (‘scope and purpose’) showed the highest score [mean = 74.4% (SD, 17%)] and Domain 5 (‘applicability’) showed the lowest score [mean = 24.3% (SD, 8.9%)]. The scores of other domains from high to low were Domain 4 [‘clarity of presentation’; mean = 73.9% (SD, 17.4%)], Domain 2 [‘stakeholder involvement’; mean = 45.9% (SD, 23.7%)], Domain 3 [‘rigor of development’; mean = 43.5% (SD, 20.4%)], and Domain 6 [‘editorial independence’; mean = 26.7% (SD, 25.0%)]. The highest item score was Item 1, and the lowest was Item 19. The average score of Item 19 was one, indicating that all 24 CPGs lack the description of Item 19 **(S5 Table)**. The ICC values in all domains and overall rating were all > 0.75, indicating that the consistency among the three researchers was relatively high **(S3 Table)**.

### 3.4 Quality of CPG recommendations

**Fig 4:**
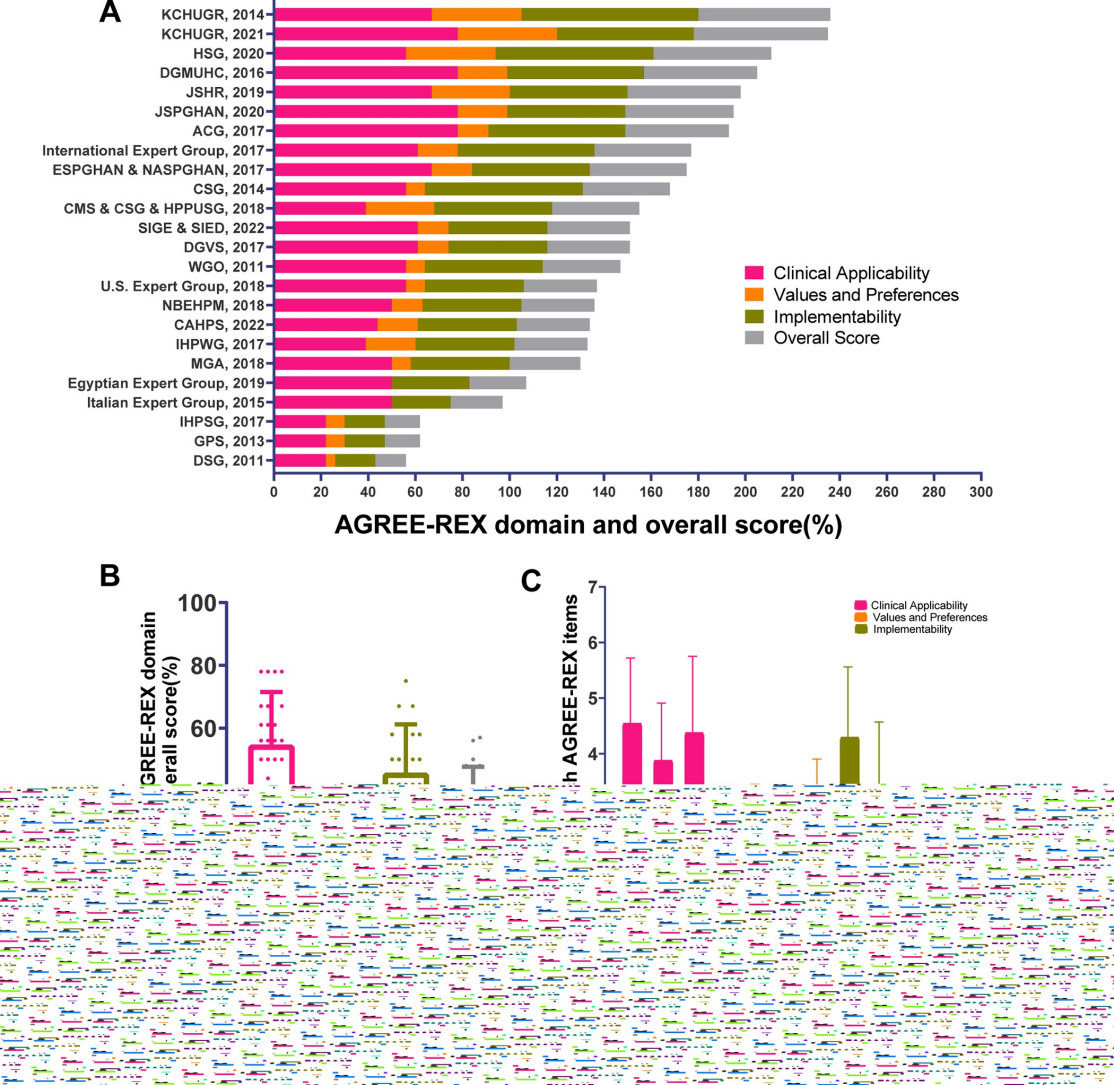
AGREE-REX scores. (A) AGREE-REX domain and overall score in each CPGs. (B)Average score of each AGREE-REX domain and overall score for all CPGs. (C) Average score of each AGREE-REX item for all CPGs. CPG, Clinical practice guideline; AGREE-REX, the Appraisal of Guidelines Research and Evaluation-Recommendations Excellence.

**Fig 4 and S6 Table** show the AGREE-REX score of the included CPGs. The mean overall score of the CPG recommendations was 35.5% (SD = 12.2%), with the highest score in the domain of ‘clinical applicability’ [mean = 54.5% (SD, 17.0%)] and the lowest score in the domain of ‘values and preferences’ [mean = 16.6% (SD, 17.9%)]. ‘Implementability’ was considered to show a moderate performance [mean = 45.6% (SD, 15.6%)]. The three items with the highest scores were Item 1, ‘evidence’; Item 2, ‘applicability to target users’ (both in the clinical applicability domain); and Item 8, ‘purpose’ (in the implementability domain). The three lowest scoring items all belonged to ‘values and preferences’: Item 4, ‘values and preferences of target users’; Item 5, ‘values and preferences of patients and populations’; and Item 6, ‘values and preferences of policy- or decision-makers.’ The contents of these items were reflected in the 22 CPGs, but not comprehensively **(S7 Table).**

### 3.5 Quality of CPG reporting

**Fig 5:**
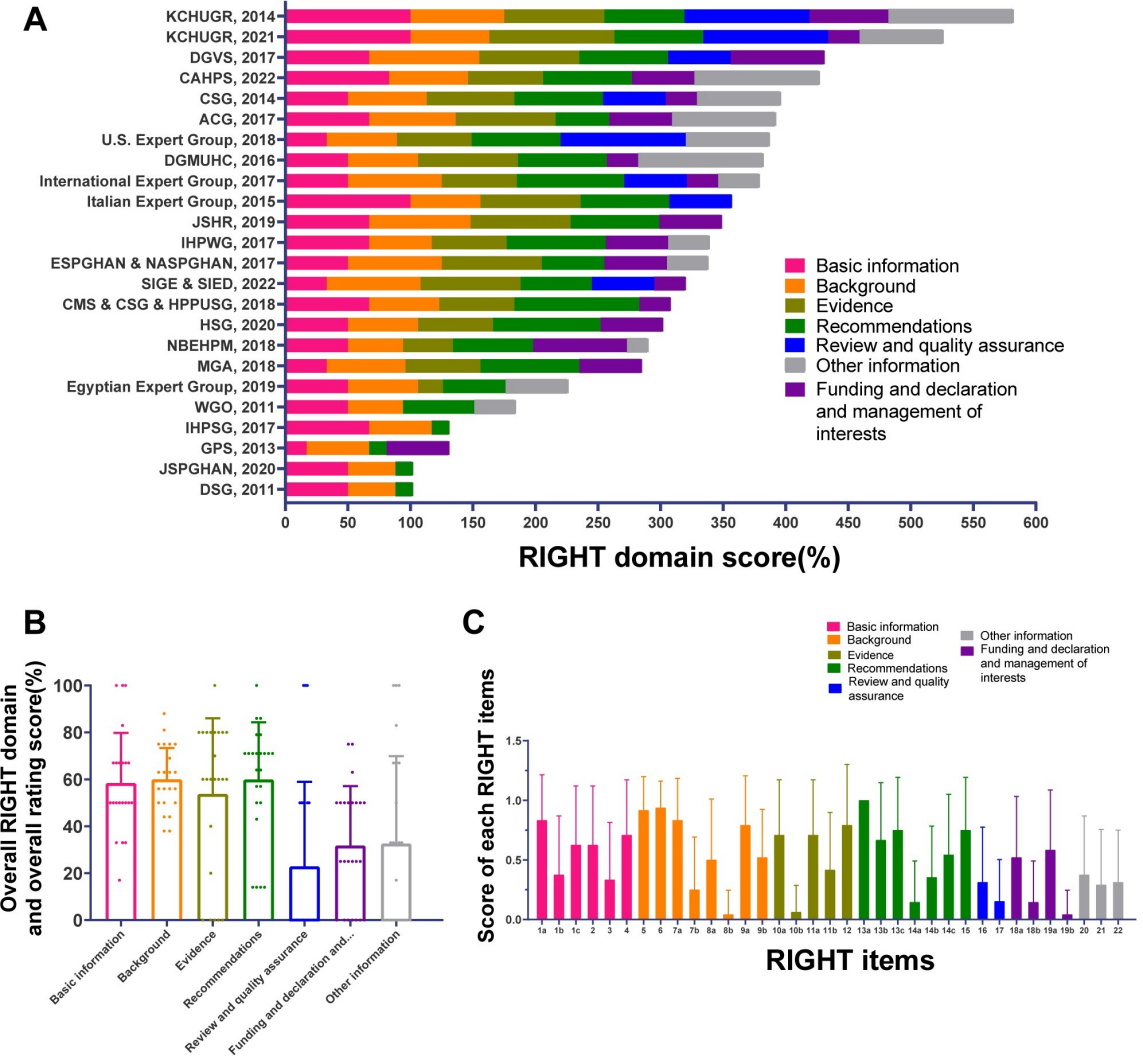
RIGHT scores. (A) RIGHT domain score in each CPGs. (B)Average score of each RIGHT domain for all CPGs. (C) Average score of each RIGHT item for all CPGs. CPG, Clinical practice guideline; RIGHT, the Reporting Items for Practice Guidelines in Healthcare.

**Fig 5 and S8 Table** show the RIGHT score of the included CPGs. It was found that among the seven domains of RIGHT, Domain 4 (‘recommendations’) had the highest reporting rate of 60.0% (SD = 24.4%), and domain 5 (‘review and quality assurance’) had the lowest reporting rate of 22.9% (SD = 36.1%). Domain 1 (‘basic information’), Domain 2 (‘background’), Domain 3 (‘evidence’), and Domain 4 (‘recommendations’) had a high reporting rate, while Domain 5 (‘review and quality assurance’), Domain 6 (‘funding and declaration and management of interests’), and Domain 7 (‘other information’) had a low reporting rate. There was a large gap between the four domains with high reporting rates and the three domains with low reporting rates.

In different domains, the quality of each item report was uneven, and the gap was obvious. The top three scoring items were Item 5, 6, and 13a, which all scored almost one. However, the lowest scoring items were Item 8b, 10b, and 19b, which all scored almost zero **(S9 Table)**.

### 3.6 Level of evidence and strength of recommendations

**Fig 6:**
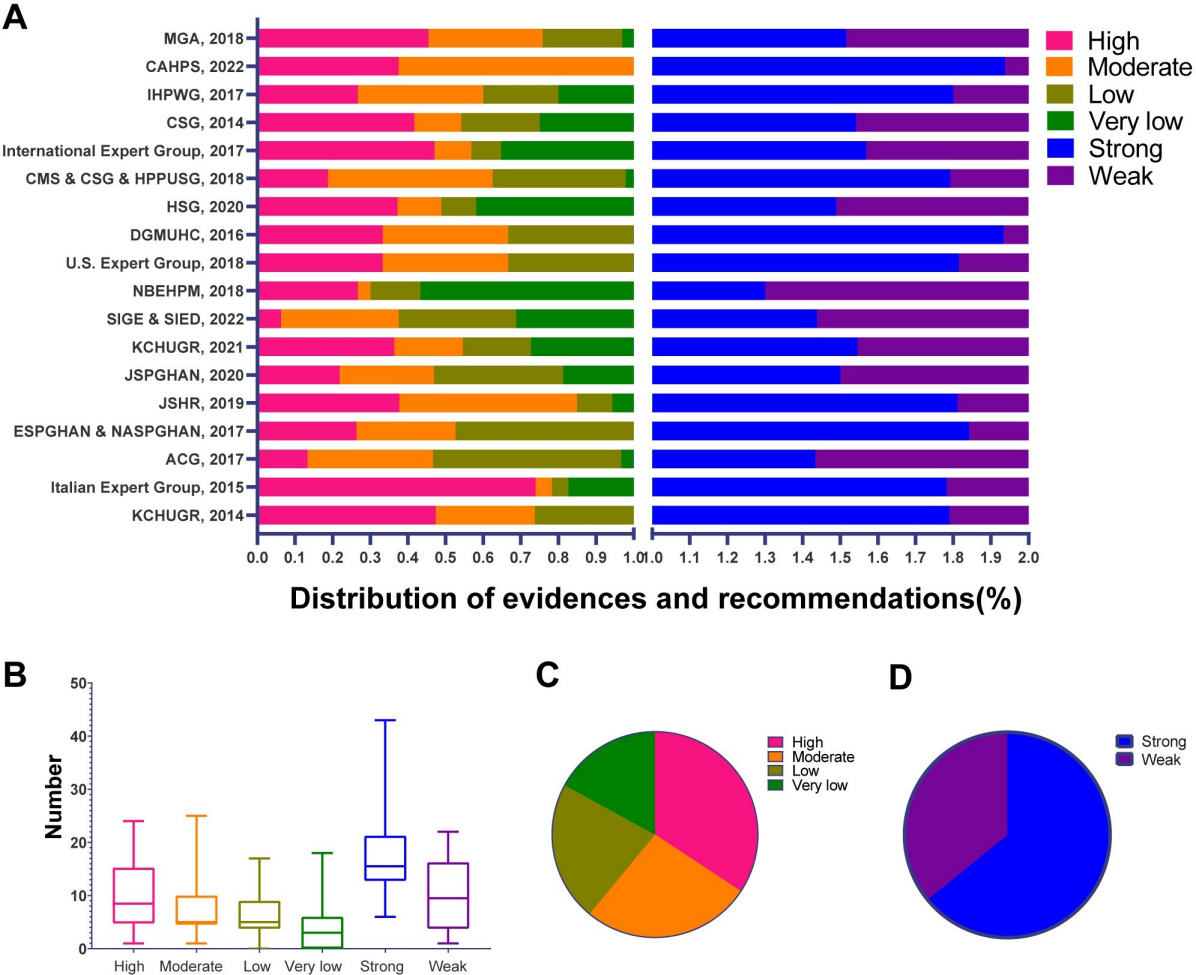
The level of evidence and the strength of recommendations. (A) Distribution of the level of evidence and strength of recommendation in each evidence-based CPGs. (B) The number of different levels of evidence and recommendations of different strengths for all evidence-based CPGs. (C) The ratio of the level of evidence. (D)The ratio of the strength of recommendations. CPG, Clinical practice guideline.

Of the 19 evidence-based CPGs, 13 used the GRADE system, four used the Oxford system and its adaptations, one used the United States Preventive Services Task Force criteria, and one did not mention the grading system **(S10 Table)**. A total of 505 recommendations were identified **(S11 Table)**. After reassessment, it was found that the distribution of the level of evidence and the strength of recommendations in each CPG was varied **(Fig 6A)**.

In the CPG of the Italian expert group [56], high-quality evidence accounted for 73.9% of all evidence and strong recommendations accounted for 78.3% of all recommendations, which was commendable for the composition of evidence and recommendations. In contrast, in the CPG of SIGE&SIED [30], only 6.3% of the evidence was of high level, while only 43.8% of the recommendations were strongly recommended **(S11 Table)**. Across all CPGs, the median numbers of high-level types of evidence and strong recommendations were 8.5 (Q1–Q3, 5.0–13.8) and 15.5 (Q1–Q3, 13.0–20.2), respectively **(S12 Table; Fig 6B).** Among the 505 identified recommendations and corresponding evidence, strong recommendations accounted for 64.1% and high-level evidence accounted for only 34.3%. At the same time, 26.7% of evidence was rated as moderate, 22% as low, and 17% as very low **(S11 Table; Fig 6C and 6D).**

### 3.7 Subgroup analyses

There were significant differences in the AGREE II overall rating between the fields of ‘version’ (updated vs. first, p = 0.004), ‘development method’ (EB vs. CB, p = 0.004), and ‘inclusion of a CPG methodologist’ (yes vs. no, p = 0.003). Notably, CPGs that were from developed countries and were based on evidence or used CPG quality tools got higher scores in each of the six domains of AGREE II than CPGs that were from developing countries and were based on consensus or did not use CPG quality tools. In addition, except for the application domain, updated CPGs scored higher than the first version in all domains **(Table 1).**

**Table 1:** AGREE II domain and overall rating scores for different subgroups of CPGs (Mean ± SD, %).

Subgroups	Statistics	Scope and purpose	Stakeholder involvement	Rigour of development	Clarity of presentation	Applicability	Editorial independence	Overall rating
All CPGs	24 (100.0%)	74.4 ± 17.1	45.9 ± 23.7	43.5 ± 20.4	73.9 ± 17.4	24.3 ± 8.9	26.7 ± 25.0	50.7 ± 17.2
Type of development organization		P = 0.626	P = 0.368	P = 0.492	P = 0.774	P = 0.078	P = 0.618	P = 0.388
Medical society	14 (58.3%)	77.3 ± 19.2	48.6 ± 25.7	45.8 ± 21.7	74.3 ± 17.6	27.3 ± 8.5	22.8 ± 19.1	50.3 ± 16.5
Expert panel	8 (33.3%)	70.8 ± 14.5	37.4 ± 12.7	36.9 ± 15.9	71.4 ± 19.8	18.6 ± 5.7	34.0 ± 35.8	47.4 ± 15.5
Government	2 (8.3%)	68.5 ± 13.4	61.5 ± 44.5	53.5 ± 31.8	81.5 ± 7.8	26.5 ± 16.3	25.0 ± 8.5	66.5 ± 31.8
Country		P = 0.010	P = 0.033	P = 0.100	P = 0.130	P = 0.082	P = 0.885	P = 0.091
Developed country	16 (66.7%)	80.4 ± 16.3	53.1 ± 25.6	48.3 ± 21.6	77.8 ± 16.6	26.6 ± 8.5	27.2 ± 23.1	54.9 ± 18.2
Developing country	8 (33.3%)	62.2 ± 11.8	31.6 ± 9.1	33.8 ± 14.3	66.2 ± 17.4	19.9 ± 8.5	25.6 ± 30.3	42.2 ± 12.2
Version		P = 0.113	P = 0.079	P = 0.005	P = 0.006	P = 0.567	P = 0.032	P = 0.004
Updated	14 (58.3%)	79.1 ± 15.2	53.1 ± 25.1	52.7 ± 16.0	81.7 ± 7.4	23.4 ± 7.7	35.8 ± 25.8	58.8 ± 13.3
First	10 (41.7%)	67.8 ± 18.2	35.9 ± 18.1	30.5 ± 19.3	63.0 ± 21.7	25.6 ± 10.7	14.0 ± 18.2	39.3 ± 16.0
Development method		P = 0.448	P = 0.077	P = 0.003	P < 0.001	P = 0.300	P = 0.155	P = 0.004
EB	18 (75.0%)	75.9 ± 15.2	50.8 ± 21.8	50.2 ± 14.6	80.2 ± 8.2	25.4 ± 8.1	30.9 ± 24.9	56.2 ± 12.7
CB	6 (25.0%)	69.7 ± 22.8	31.2 ± 24.6	23.2 ± 22.9	55.0 ± 24.4	21.0 ± 11.0	14.0 ± 22.8	34.2 ± 19.6
Used CPG quality tool		P = 0.993	P = 0.017	P = 0.030	P = 0.111	P = 0.035	P = 0.298	P = 0.052
Yes	15 (62.5%)	74.6 ± 15.8	54.3 ± 22.2	50.6 ± 15.8	79.0 ± 8.5	26.7 ± 8.2	29.7 ± 21.3	56.6 ± 13.9
No	7 (29.2%)	74.3 ± 18.2	38.3 ± 17.2	36.1 ± 23.6	62.4 ± 27.4	17.4 ± 5.9	27.9 ± 32.9	43.7 ± 20.2
Not stated	2 (8.3%)	73.0 ± 35.4	9.5 ± 7.8	15.5 ± 2.1	76.0 ± 12.7	30.5 ± 13.4	0.0 ± 0.0	30.5 ± 3.5
Included CPG methodologist		P = 0.262	P = 0.018	P = 0.020	P = 0.533	P = 0.366	P = 0.364	P = 0.003
Yes	3 (12.5%)	89.7 ± 11.1	80.3 ± 22.8	72.7 ± 17.2	84.7 ± 7.8	30.7 ± 6.7	46.3 ± 13.9	79.7 ± 11.4
No	12 (50.0%)	71.9 ± 13.4	41.8 ± 15.7	40.6 ± 15.7	73.0 ± 16.8	22.4 ± 9.2	23.6 ± 28.6	46.8 ± 12.0
Not stated	9 (37.5%)	72.6 ± 21.5	40.0 ± 25.1	37.6 ± 20.2	71.6 ± 20.5	24.8 ± 8.9	24.3 ± 21.6	46.2 ± 16.2
Funding sources		P = 0.823	P = 0.272	P = 0.143	P = 0.444	P = 0.462	P = 0.320	P = 0.388
Yes	13 (54.2%)	75.7 ± 18.4	52.0 ± 24.6	50.8 ± 19.4	73.9 ± 18.0	23.5 ± 7.6	32.7 ± 23.7	55.1 ± 17.7
No	7 (29.2%)	70.9 ± 13.3	43.6 ± 16.7	36.6 ± 19.3	68.7 ± 19.3	23.0 ± 10.3	24.6 ± 28.2	46.9 ± 17.3
Not stated	4 (16.7%)	76.2 ± 22.2	30.2 ± 28.5	31.5 ± 19.8	83.0 ± 11.0	29.5 ± 11.0	11.0 ± 22.0	43.0 ± 15.3
Scope		P = 0.040	P = 0.381	P = 0.116	P = 0.224	P = 0.258	P = 0.552	P = 0.111
Treatment	8 (33.3%)	81.6 ± 11.0	50.8 ± 24.1	51.8 ± 12.6	82.4 ± 6.1	23.5 ± 7.5	20.8 ± 15.1	58.5 ± 13.6
Diagnosis, treatment	13 (54.2%)	66.7 ± 18.2	40.0 ± 23.3	35.6 ± 22.9	68.7 ± 21.1	23.0 ± 9.5	27.4 ± 31.0	43.9 ± 17.9
Diagnosis, treatment, prevention	3 (12.5%)	88.3 ± 8.0	58.7 ± 24.2	55.3 ± 14.0	74.0 ± 15.4	32.3 ± 7.4	39.7 ± 15.9	59.0 ± 14.1
Year		P = 0.941	P = 0.813	P = 0.611	P = 0.994	P = 0.287	P = 0.679	P = 0.673
≤2016	8 (33.3%)	74.8 ± 22.4	44.2 ± 29.2	40.4 ± 25.9	73.9 ± 23.0	27.1 ± 8.1	23.6 ± 24.9	48.5 ± 21.0
>2016	16 (66.7%)	74.2 ± 14.6	46.8 ± 21.4	45.0 ± 17.8	73.9 ± 14.8	22.9 ± 9.2	28.2 ± 25.8	51.8 ± 15.7

CPG, clinical practice guideline; EB, evidence-based; CB, consensus-based; AGREE, Appraisal of Guidelines for Research and Evaluation.

The overall score of AGREE-REX showed significant differences in the fields of ‘development method’ (EB vs. CB, p = 0.001) and ‘included a CPG methodologist’ (yes vs. no, p = 0.008). Among different stratified criteria, CPGs that were established by the government of a developed country and were based on evidence, used a CPG quality tool, included a CPG methodologist, had funding sources, and were published after 2016 had a higher overall score **(Table 2).**

**Table 2:** AGREE-REX domain and overall scores for different subgroups of CPGs (Mean ± SD, %).

Subgroups	Statistics	Clinical applicability	Values and preferences	Implementability	Overall score
All CPGs	24 (100.0%)	54.5 ± 17.0	16.6 ± 11.9	45.6 ± 15.6	35.5 ± 12.2
Type of development organization		P = 0.873	P = 0.031	P = 0.433	P = 0.267
Medical society	14 (58.3%)	55.3 ± 17.8	18.6 ± 11.5	48.4 ± 16.6	37.2 ± 12.1
Expert panel	8 (33.3%)	52.1 ± 15.5	9.4 ± 7.4	39.6 ± 14.5	30.2 ± 10.5
Government	2 (8.3%)	58.5 ± 27.6	31.5 ± 14.8	50.0 ± 11.3	44.0 ± 18.4
Country		P = 0.025	P = 0.307	P = 0.423	P = 0.091
Developed country	16 (66.7%)	59.9 ± 17.3	18.4 ± 12.9	47.4 ± 16.5	38.4 ± 13.3
Developing country	8 (33.3%)	43.8 ± 10.6	13.0 ± 9.1	41.9 ± 14.1	29.5 ± 7.2
Version		P = 0.055	P = 0.159	P = 0.233	P = 0.068
Updated	14 (58.3%)	60.1 ± 13.4	19.5 ± 12.1	48.9 ± 11.6	39.3 ± 10.2
First	10 (41.7%)	46.7 ± 19.1	12.5 ± 10.8	41.0 ± 19.8	30.1 ± 13.3
Development method		P = 0.006	P = 0.017	P = 0.001	P = 0.001
EB	18 (75.0%)	59.7 ± 13.1	19.8 ± 11.9	51.0 ± 12.0	39.8 ± 9.8
CB	6 (25.0%)	38.8 ± 18.8	6.8 ± 4.4	29.3 ± 14.5	22.5 ± 9.7
Used CPG quality tool		P = 0.183	P = 0.105	P = 0.066	P = 0.052
Yes	15 (62.5%)	59.1 ± 13.3	20.4 ± 12.9	51.2 ± 12.9	39.9 ± 10.4
No	7 (29.2%)	49.1 ± 20.8	11.4 ± 6.9	37.0 ± 15.7	29.6 ± 12.2
Not stated	2 (8.3%)	39.0 ± 24.0	6.0 ± 2.8	33.5 ± 23.3	23.0 ± 14.1
Included CPG methodologist		P = 0.079	P = 0.015	P = 0.039	P = 0.008
Yes	3 (12.5%)	74.3 ± 6.4	33.7 ± 11.2	63.7 ± 9.8	53.7 ± 4.9
No	12 (50.0%)	53.3 ± 14.2	15.8 ± 10.2	46.7 ± 14.0	34.9 ± 9.5
Not stated	9 (37.5%)	49.4 ± 19.1	12.0 ± 9.9	38.1 ± 14.9	30.1 ± 11.9
Funding sources		P = 0.591	P = 0.617	P = 0.295	P = 0.429
Yes	13 (54.2%)	57.8 ± 16.7	18.2 ± 10.7	49.5 ± 14.5	38.1 ± 11.4
No	7 (29.2%)	51.7 ± 17.6	16.7 ± 13.3	44.1 ± 16.4	34.3 ± 12.5
Not stated	4 (16.7%)	48.8 ± 19.2	11.2 ± 14.9	35.5 ± 17.1	29.0 ± 15.1
Scope		P = 0.029	P = 0.390	P = 0.512	P = 0.115
Treatment	8 (33.3%)	66.1 ± 15.1	20.0 ± 12.2	50.9 ± 11.2	42.0 ± 10.1
Diagnosis, treatment	13 (54.2%)	46.7 ± 15.5	13.5 ± 11.9	42.5 ± 19.1	30.8 ± 12.7
Diagnosis, treatment, prevention	3 (12.5%)	57.3 ± 11.9	21.0 ± 10.6	44.7 ± 4.6	38.0 ± 8.9
Year		P = 0.657	P = 0.655	P = 0.879	P = 0.689
≤2016	8 (33.3%)	52.2 ± 20.6	15.0 ± 14.1	44.9 ± 22.6	34.0 ± 16.2
>2016	16 (66.7%)	55.6 ± 15.6	17.4 ± 11.1	45.9 ± 11.7	36.2 ± 10.2

CPG, clinical practice guideline; EB, evidence-based; CB, consensus-based; AGREE-REX: Appraisal of Guidelines for Research and Evaluation-Recommendation Excellence.

In the subgroup analyses of the RIGHT results, it was found the CPGs that were based on evidence and used a CPG quality tool tended to perform better in the RIGHT domains ‘evidence’ and ‘recommendation’ **(Table 3).** The number of very low-level evidence items and weak recommendations had significant differences in the field of ‘used a CPG quality tool’ (yes vs. no, p < 0.01) **(S13 Table).**

**Table 3:** RIGHT domain and overall rating scores for different subgroups of CPGs (Mean ± SD, %).

Subgroups	Statistics	Basic information	Background	Evidence	Recommendations	Review and quality assurance	Funding and declaration and management of interests	Other information
All CPGs	24 (100.0%)	58.4 ± 21.5	60.0 ± 13.4	53.8 ± 32.3	60.0 ± 24.4	22.9 ± 36.1	31.8 ± 25.3	32.6 ± 37.3
Type of development organization		P = 0.219	P = 0.651	P = 0.501	P = 0.545	P = 0.509	P = 0.415	P = 0.785
Medical society	14 (58.3%)	57.2 ± 20.4	62.2 ± 15.9	52.1 ± 35.1	55.9 ± 26.7	17.9 ± 31.7	36.6 ± 24.3	29.7 ± 40.4
Expert panel	8 (33.3%)	54.1 ± 21.5	57.0 ± 9.1	50.0 ± 28.3	63.2 ± 22.5	25.0 ± 37.8	21.9 ± 28.1	33.4 ± 36.8
Government	2 (8.3%)	83.5 ± 23.3	56.5 ± 9.2	80.0 ± 28.3	75.0 ± 5.7	50.0 ± 70.7	37.5 ± 17.7	50.0 ± 24.0
Country		P = 1.000	P = 0.267	P = 0.433	P = 0.402	P = 0.111	P = 0.732	P = 0.946
Developed country	16 (66.7%)	58.4 ± 24.4	62.2 ± 15.3	57.5 ± 35.7	56.9 ± 24.1	31.2 ± 40.3	30.5 ± 25.4	32.2 ± 38.7
Developing country	8 (33.3%)	58.4 ± 15.5	55.6 ± 7.2	46.2 ± 24.5	66.0 ± 25.4	6.2 ± 17.7	34.4 ± 26.5	33.4 ± 36.8
Version		P = 0.107	P = 0.132	P = 0.028	P = 0.109	P = 0.141	P = 0.131	P = 0.798
Updated	14 (58.3%)	64.4 ± 22.6	63.5 ± 14.5	65.7 ± 24.1	66.7 ± 20.6	32.1 ± 42.1	38.4 ± 26.2	30.9 ± 35.1
First	10 (41.7%)	50.0 ± 17.5	55.1 ± 10.5	37.0 ± 35.9	50.5 ± 27.1	10.0 ± 21.1	22.5 ± 21.9	35.0 ± 41.9
Development method		P = 0.289	P = 0.239	P < 0.001	P = 0.004	P = 0.262	P = 0.228	P = 0.158
EB	18 (75.0%)	61.1 ± 22.2	61.9 ± 11.7	66.1 ± 21.5	67.7 ± 18.8	27.8 ± 39.2	35.4 ± 22.0	38.9 ± 39.6
CB	6 (25.0%)	50.2 ± 18.3	54.3 ± 17.6	16.7 ± 32.0	36.7 ± 25.7	8.3 ± 20.4	20.8 ± 33.2	13.8 ± 22.1
Used CPG quality tool		P = 0.360	P = 0.056	P < 0.001	P = 0.016	P = 0.427	P = 0.172	P = 0.196
Yes	15 (62.5%)	63.3 ± 23.8	63.8 ± 9.2	72.7 ± 12.2	70.3 ± 13.8	30.0 ± 41.4	35.9 ± 19.5	43.3 ± 41.7
No	7 (29.2%)	50.1 ± 16.7	57.3 ± 17.9	28.6 ± 32.4	44.7 ± 30.6	14.3 ± 24.4	32.1 ± 34.5	14.3 ± 20.2
Not stated	2 (8.3%)	50.0 ± 0.0	41.0 ± 4.2	0.0 ± 0.0	35.5 ± 30.4	0.0 ± 0.0	0.0 ± 0.0	16.5 ± 23.3
Included CPG methodologist		P = 0.081	P = 0.815	P = 0.171	P = 0.715	P = 0.059	P = 0.677	P = 0.007
Yes	3 (12.5%)	83.3 ± 28.9	64.7 ± 9.6	86.7 ± 11.5	68.7 ± 4.0	66.7 ± 57.7	37.7 ± 21.9	89.0 ± 19.1
No	12 (50.0%)	52.8 ± 14.0	58.9 ± 12.0	49.2 ± 28.1	61.2 ± 26.6	20.8 ± 33.4	27.1 ± 24.9	30.6 ± 34.0
Not stated	9 (37.5%)	57.6 ± 23.8	59.9 ± 16.8	48.9 ± 37.6	55.4 ± 26.0	11.1 ± 22.0	36.1 ± 28.3	16.6 ± 28.7
Funding sources		P = 0.429	P = 0.529	P = 0.587	P = 0.790	P = 0.792	P = 0.010	P = 0.318
Yes	13 (54.2%)	60.3 ± 24.1	62.8 ± 14.2	59.2 ± 30.1	59.8 ± 23.3	26.9 ± 38.8	45.2 ± 21.4	41.0 ± 38.3
No	7 (29.2%)	50.0 ± 13.9	57.9 ± 7.9	51.4 ± 30.2	64.1 ± 27.8	21.4 ± 39.3	17.9 ± 18.9	31.0 ± 41.4
Not stated	4 (16.7%)	66.8 ± 23.6	54.8 ± 19.0	40.0 ± 46.2	53.2 ± 27.0	12.5 ± 25.0	12.5 ± 25.0	8.2 ± 16.5
Scope		P = 0.104	P = 0.028	P = 0.093	P = 0.564	P = 0.948	P = 0.138	P = 0.811
Treatment	8 (33.3%)	66.8 ± 21.8	61.0 ± 12.3	67.5 ± 30.1	63.2 ± 26.9	25.0 ± 37.8	25.0 ± 18.9	39.5 ± 39.8
Diagnosis, treatment	13 (54.2%)	50.0 ± 20.5	55.4 ± 11.3	40.8 ± 32.3	55.4 ± 25.6	23.1 ± 38.8	29.8 ± 27.8	28.2 ± 33.6
Diagnosis, treatment, prevention	3 (12.5%)	72.3 ± 9.2	77.3 ± 12.9	73.3 ± 11.5	71.0 ± 0.0	16.7 ± 28.9	58.3 ± 14.4	33.3 ± 57.7
Year		P = 0.740	P = 0.594	P = 0.603	P = 0.419	P = 0.846	P = 0.491	P = 0.661
≤2016	8 (33.3%)	60.5 ± 28.0	57.9 ± 14.7	48.8 ± 40.5	54.1 ± 25.3	25.0 ± 37.8	26.6 ± 25.5	37.5 ± 45.2
>2016	16 (66.7%)	57.3 ± 18.3	61.1 ± 13.0	56.2 ± 28.5	62.9 ± 24.2	21.9 ± 36.4	34.4 ± 25.6	30.2 ± 34.0

CPG, clinical practice guideline; EB, evidence-based; CB, consensus-based; RIGHT: Reporting Items for Practice Guidelines in Healthcare.

### 3.8 Correlations among the AGREE II, AGREE-REX, and RIGHT domains

**Fig 7:**
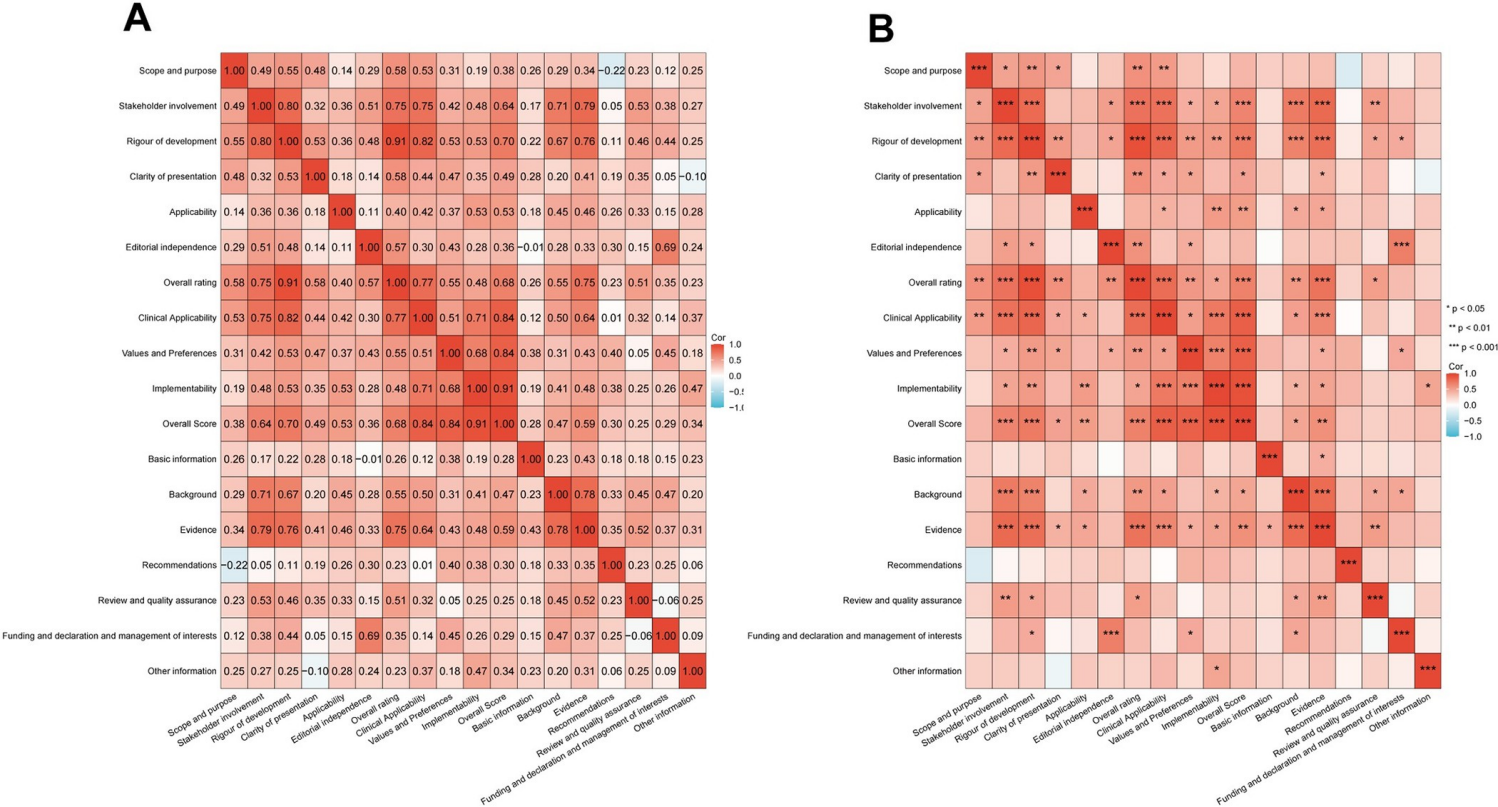
Correlations among the AGREE II, AGREE-REX and RIGHT domains. (A) Heat map of Pearson correlation coefficient between AGREE II, AGREE-Rex, and RIGHT domains. (B) Heat map of P value for correlation between AGREE II, AGREE-Rex, and RIGHT domains, *: p < 0.05, **: p < 0.01, ***: p < 0.001. AGREE II, the Appraisal of Guidelines for Research and Evaluation II; AGREE-REX, the Appraisal of Guidelines Research and Evaluation-Recommendations Excellence; RIGHT, the Reporting Items for Practice Guidelines in Healthcare.

Most of the AGREE II, AGREE-REX, and RIGHT domains were positively correlated with each other **(Fig 7)**. There was a high positive correlation between the ‘overall rating’ of AGREE II and the domain ‘rigor of development’ (r = 0.91). In addition, the ‘overall score’ of AGREE-REX exhibited a high positive correlation with the domains ‘implementability’ (r = 0.91), ‘values and preferences’ (r = 0.84), and ‘rigor of development’ (r = 0.84). There was also a strong positive correlation between ‘clinical applicability’ and ‘rigor of development’ (r = 0.82). Meanwhile, ‘rigor of development’ showed a high positive correlation with ‘stakeholder involvement’ (r = 0.80). ‘Evidence’ was positively associated with ‘stakeholder involvement’ (r = 0.79), ‘background’ (r = 0.78), ‘rigor of development’ (r = 0.76), and ‘overall rating’ (r = 0.75). All the above mentioned had significant differences (p < 0.001).

## 4 Discussion

Overall, the methodological quality, recommendation quality, and reporting quality of CPGs for HP infection were generally low. Moreover, only three of the 24 CPGs were of high quality. The quality of the CPGs was highly heterogeneous and the same CPGs often had varied scores for different domains. Meanwhile, there was a significant correlation between the AGREE II, AGREE-REX, and RIGHT domains. Overall, 19 CPGs were considered to be evidence-based; however, the CPGs lacked high-quality evidence to support the recommendations. Therefore, first-class research is needed to minimize the large evidence gap. It was also found that specific factors significantly affect the quality of the CPGs, and these should be taken into account in decision making during the CPGs development process.

A total of 24 CPGs were retrieved from > 12 countries, of which five produced ≥ 2 CPGs. However, after evaluating the included CPGs using the AGREE II tool, it was found that there was a large gap in the quality of each CPG, and the overall quality of the 24 CPGs was not high. Only three CPGs [33, 54, 55] were evaluated as high quality. An unfortunate phenomenon is that the number of CPGs is high but the number of high-quality CPGs is low. A high number of low-quality CPGs will not provide more clinical options, but may produce some negative results. Spending resources on low-quality CPGs and ineffective treatment recommendations is wasteful and leaves users confused [41].

There is a pressing need for further improvement of the clinical applicability of these CPGs, which would greatly facilitate physicians in applying the recommendations within their clinical practice. In the evaluation conducted by AGREE II, it was observed that domain 5 ‘applicability’ received the lowest score. Interestingly, even the three high-quality CPGs exhibited shallow scores in domain 5, and few domain-related content was described in the CPGs. This is a significant concern that not all healthcare facilities can meet the CPGs’ requirements, potentially impeding recommendations’ effective implementation [57, 58].

To make the CPGs more effective, additional materials are needed to improve generalization and implementation [37]. A point of interest is that for each AGREE II item, the score of Item 19 (the guideline provides advice or tools to help put the recommendations into practice) of each CPG was zero. Therefore, it is a serious defect that all CPGs had missing content for Item 19, as this may lead to difficulties in the promotion and use of the CPGs.

Among three high quality CPGs, the CPG of DGMUHC [54] suggests that the primary initial treatment for HP infection should be non-bismuth quadruple therapy and traditional bismuth quadruple therapy, with a recommended treatment duration of 14 days to ensure a high rate of successful eradication. This CPG also recommends PPI triple therapy only in regions where the prevalence of clarithromycin resistance is below 15% or where local eradication rates are consistently high. The CPG further states that studies have demonstrated a decline in the efficacy of PPI triple therapy for eradication rates over time when compared to non-bismuth and bismuth quadruple therapy [59]. The CPG provided by KCHUGR [55] suggests that quadruple therapy or bismuth-containing quadruple therapy can be considered as an alternative treatment for HP infection. However, the primary eradication approach for HP infection, as outlined in the CPG, is PPI triple therapy. The CPG of DGVS [33] provides a greater number of prevention recommendations compared to the previous two CPGs. This holds significant reference value in terms of preventing and reducing the likelihood of transmission of HP. Additionally, there are numerous accounts regarding the diagnostic methods and indications for the treatment of HP. This CPG suggests bismuth-containing quadruple therapy or a concomitant quadruple therapy as the preferred initial treatment option in cases where there is a high probability of primary clarithromycin resistance. Conversely, in situations where primary clarithromycin resistance is less probable, standard triple therapy or bismuth-containing quadruple therapy should be considered. Despite minor variations in the recommendations for HP treatment, three high quality CPGs concur that triple therapy or quadruple therapy should be employed. In contrast to two other high-quality CPGs, the CPG of DGVS [33] is not grounded in evidence-based medical research. While experts’ clinical experience can offer valuable insights, the strength of recommendations relies on the level of evidence employed to substantiate them, and the development of CPG is more dependent on the growing evidence [60].

CPGs in the process of developing need to pay more attention to the values and preferences, and how to effectively incorporate the views of target users, patients, and developers. The recommendations in the CPGs were evaluated through AGREE-REX, and the score of ‘values and preferences’ was the lowest by far compared to that of the other two fields. Almost every CPG had a low score in this field, which is worthy of attention. Values and preferences undoubtedly influence a person’s judgment, thus, likely influence the CPG development team members’ recommendations. Regarding values and preferences, a systematic review assessed how guidance documents that develop CPGs address the inclusion of patient perspectives and found that although most institutions recommended the inclusion of patients and their perspectives when developing CPGs, little detail is typically provided about how to do this [61].

Understanding the purpose of the RIGHT checklist is necessary to assist CPG developers in reporting CPGs, to support peer reviewers in considering CPG reports, and to assist clinicians in understanding and implementing CPGs. Therefore, it is important to improve the quality of CPG reporting during the production or revision of CPGs in the future [62]. Among the seven domains of the RIGHT scale, Domain 4 (‘recommendations’) had the highest reporting rate, while Domain 5 (‘rationale/explanation for recommendations’) had the lowest reporting rate. Domain 5 was the lowest because too few CPGs reported in the domain of review or quality assurance, of which only four CPGS reported Item 17 (‘quality assurance’). In the overall high scoring Domain 2 and 3, one item from each had a very poor score, which affected the score of the domain. Among the items in the ‘evidence’ domain 10b, only two CPGs were ‘reported’ and ‘partially reported,’ respectively. However, outcome selection is very important in the formulation of the PICO (patient, intervention, control, and outcome) question because it affects the balance of benefits and harms on which the proposal is based, and readers need to know how and why certain outcomes are selected [63, 64]. In total, there were 35 items in seven domains. Almost every CPG had many items that were not reported, and the content of many reported items was not elaborated on in detail.

Furthermore, in addition to focusing on domains where CPGs are performing badly, CPG developers should consider the inclusion of high-quality evidence. While the goal of developing CPGs is to create a safer medical system, the strength of their recommendations depends on the level of evidence used to support them [65]. After re-grading the level of evidence and the strength of recommendations using the GRADE system, here, it was found that although the number of strong recommendations was high, only 111 of 173 strong recommendations were based on high-quality evidence, which is paradoxical **(S8 Table)**. Consistency between the level of evidence and the strength of recommendations is important, but if the link is inconclusive, it will violate a key principle of evidence-based medicine and may run the risk of being misleading [66–69]. In addition, inappropriately strong recommendations may limit future randomized trials that can produce higher-quality evidence [70]. More first-class research is needed to support current recommendations. Meanwhile, the distribution of evidence level and strength of recommendations varied greatly among different CPGs. The CPGs of an Italian expert group [56] **(Fig 6A)** showed the best performance in terms of distribution of evidence and recommendations, which is a paradigm that could be referred to by CPG developers.

As many of the improvements in the CPG development process have become the norm, the quality of the guidelines has improved over time, but there remains scope for further improvement. The analysis of the correlation among the domains of AGREE II, AGREE-REX, and RIGHT revealed that there is a close relationship between the methodology, recommendations, and reporting quality. High-quality CPGs should demonstrate strength in these three dimensions. Many aspects of CPG development need to be improved. In the subgroup analysis of the CPG quality evaluation results, it was found that CPGs that were updated, evidence-based, and had a methodologist involved tended to show a higher score for each domain. Not only that, but the CPGs developed by government agencies were also better quality than those developed by other agencies, indicating the importance of establishing a system of dissemination, collection, and implementation of CPGs at a national level [71]. In addition, the quality of CPGs for HP infection from developed countries was higher. Although the management of HP infection has improved in developing countries, there remains a gap between actual practice and CPGs [72]. Prior to the release of CPGs, CPG organizations should evaluate them by using quality assessment tools and describe the quality of the guidelines, which could help improve their reliability. A funded CPG often means more resources are available and the quality of the CPGs will be higher. The use of a CPG quality tool is also beneficial for improving the structure of evidence and recommendations.

In this study, AGREEII, RIGHT and AGREE-REX are all tools for assessing the quality of CPGs. However, they focus on different dimensions, where AGREEII focuses on the methodological quality of CPGs, RIGHT emphasizes the reporting quality of CPGs reports, and AGREE-REX focuses on the quality of recommendations. Therefore, we used AGREE II, RIGHT and AGREE-REX to establish a more comprehensive and multi-level evaluation framework for CPGs, which was helpful to reveal the potential defects and room for improvement in the CPGs. Future guideline development can avoid the same methodological issues and improve the content that needs to be reported, which will help promote the transparency and standardization of guideline development. By assessing the quality of existing guidelines, physicians and clinical practitioners can also already be aware of the relevant information and quality of CPGs to a certain extent. The use of high-quality CPGs in clinical practice can furnish clinicians with robust direction to make more informed decisions and enhance the standard of patient care. Future CPG evaluation studies can also integrate the three evaluation tools to evaluate the quality of CPGs in a more comprehensive way.

The main advantage of this study is that three tools, AGREEII, RIGHT, and AGREE-REX, were used to evaluate the included CPGs in a comprehensive way to allow the identification of possible problems from different aspects and fields as well as improve and optimize new CPGs in the future. In addition, each researcher received relevant training to ensure the validity and reliability of the CPG assessment. Apart from the quality assessment of the included CPGs, the evidence and recommendations of the CPGs were also analyzed, and subgroup analysis was conducted to explore other factors affecting the quality of the CPGs. Moreover, we have incorporated a more concise approach to presentation, exemplified by the utilization of network diagrams and color coding. These visual aids effectively emphasized the research outcomes, rendering crucial information more conspicuous and easily comprehensible, which expedited readers’ comprehension and enabled them to accurately discern the strengths of the findings and identify domains for enhancement.

In terms of limitations, although a systematic literature search was performed, it is possible that not all CPGs were identified, and some eligible CPGs may have been missed. Moreover, only CPGs in English were included, limiting the number used in this study. Therefore, there may be CPGs available in other languages that were not identified. Additionally, the evaluation of CPGs using AGREE II, RIGHT, and AGREE-REX was subjective, although each researcher provided independent comments and reached a consensus with one another, and ICCs showed that the evaluation results were highly consistent and reliable. Finally, although the identified grading systems have similar frameworks, there are differences, and using the GRADE system for re-grading may result in a certain level of bias.

## 5 Conclusion

The quality of CPGs for HP infection was inconsistent, and the overall level of each field was also low. Almost no CPGs took into account the methodological, reporting, and recommendation quality collectively. The quality of CPGs for HP infection was inconsistent, and the overall level of each field was also low. Almost no CPGs took into account the methodological, reporting, and recommendation quality collectively. After evaluation, there were three CPGs with high methodological quality that most effectively fulfilled the AGREE II criteria [33, 54, 55], which could serve as valuable guidance for future clinical practice or as preferred CPGs among clinicians. In addition, the development of CPGs should ensure consistency in the level of evidence and strength of recommendations and incorporate high-quality evidence as much as possible. High-quality studies are needed to minimize the evidence gap. More high-quality CPGs need to be developed in a rigorous, internationally collaborative, and transparent manner in future to assist clinicians, policy-makers, patients, and patients’ families with making informed decisions and taking appropriate actions for effective treatment.

## Supporting information

S1 File PRISMA checklist. (DOCX)

S2 File Search strategy. (DOCX)

S1 Table Characteristics of included CPGs. (DOCX)

S2 Table Descriptive statistics of characteristics of included CPGs. (DOCX)

S3 Table Inter-rater reliability for AGREE Ⅱ domain and overall rating. (DOCX)

S4 Table AGREE II domain scores and overall assessment of included CPGs. (DOCX)

S5 Table Overall mean (SD) scores for each AGREE II item of included CPGs. (DOCX)

S6 Table AGREE-REX domain and overall scores of included CPGs. (DOCX)

S7 Table Overall mean (SD) scores for each AGREE-REX item of included CPGs. (DOCX)

S8 Table RIGHT domain scores of included CPGs. (DOCX)

S9 Table Overall mean (SD) scores for each RIGHT item of included CPGs. (DOCX)

S10 Table Grading systems used and the distribution of the level of evidence and strength of recommendation among included CPGs. (DOCX)

S11 Table Distribution of the level of evidence and strength of recommendation among evidence-based CPGs when GRADE system are used uniformly. (DOCX)

S12 Table Overall mean (SD) and median (Q1–Q3) of the number of level of evidence and strength of recommendation. (DOCX)

S13 Table The number of level of evidence and strength of recommendation for different subgroups of evidence-based CPGs (Mean ± SD, %). (DOCX)
