# Supplementary material for: Quality appraisal of clinical guidelines for Helicobacter pylori infection and systematic analysis of the level of evidence for recommendations
Source: PLoS One. 2024 Apr 10;19(4):e0301006. doi: 10.1371/journal.pone.0301006 (PMC11006150; doi:10.1371/journal.pone.0301006)
Supplement: S2 File — (DOCX) [file pone.0301006.s002.docx]

**Supplementary File 2.** The specific search strategy in scientific databases and online guideline libraries

Pubmed

| #1 "Helicobacter pylori"[Mesh] |
| --- |
| #2 (((((((helicobacter pylori*[Title/Abstract]) OR (Campylobacter* pylori*[Title/Abstract])) OR (h pylori[Title/Abstract])) OR (Helicobacter[Title/Abstract])) OR (campylobacter[Title/Abstract])) OR (pylori*[Title/Abstract])) OR (Helicobacter pylori infection[Title/Abstract])) OR (Helicobacter infection[Title/Abstract]) |
| #3 #1 OR #2 |
| #4 "Guidelines as Topic"[Mesh] |
| #5 Guideline[Publication Type] |
| #6 (((((guideline*[Title/Abstract]) OR (consensus[Title/Abstract])) OR (recommendation*[Title/Abstract])) OR (guidance[Title/Abstract])) OR (standard[Title/Abstract])) OR (statement*[Title/Abstract]) |
| #7 #4 OR #5 OR #6 |
| #8 #3 AND #7 |
| #9 #8 Filters: from 2011 - 2022 |
| #10 #9 Filters: English, from 2011 - 2022 |

#10 Result: 2,897

Embase

| #1. 'helicobacter pylori'/exp |
| --- |
| #2. 'helicobacter pylori*' OR 'campylobacter* pylori*' OR 'h pylori' OR 'helicobacter' OR 'campylobacter' OR 'pylori*' OR 'helicobacter pylori infection' OR 'helicobacter infection':ab,ti |
| #3. #1 OR #2 |
| #4. 'practice guideline'/exp |
| #5. 'practice guideline'/exp OR 'guideline*' OR 'recommendation':ab,ti |
| #6. #4 OR #5 |
| #7. #3 AND #6 |
| #8. #3 AND #6 AND [2011-2022]/py |
| #9. #3 AND #6 AND [2011-2022]/py AND [english]/lim |

Result: 2,350

Cochrane Library

| #1 MeSH descriptor: [Helicobacter pylori] explode all trees |
| --- |
| #2 (‘helicobacter pylori*’ or ‘Campylobacter* pylori*’ or ‘h pylori’ or ‘Helicobacter’ or ‘campylobacter’ or ‘pylori*’ or ‘Helicobacter pylori infection’ or ‘Helicobacter infection’):ti,ab,kw |
| #3 MeSH descriptor: [Guidelines as Topic] explode all trees |
| #4 (‘guideline*’ or ‘consensus’ or ‘recommendation*’ or ‘guidance’ or ‘standard’ or ‘statement*’):ti,ab,kw |
| #5 #1 or #2 |
| #6 #3 or #4 |
| #7 #5 and #6with Cochrane Library publication date from Jan 2011 to Oct 2022 |

Result:1,108

CINAHL

| S1 (MH "Helicobacter Pylori") |
| --- |
| S2 (MH "Helicobacter Pylori") OR ( ‘helicobacter pylori*’ or ‘Campylobacter* pylori*’ or ‘h pylori’ or ‘Helicobacter’ or ‘campylobacter’ or ‘pylori*’ or ‘Helicobacter pylori infection’ or ‘Helicobacter infection’ ) |
| S3 (MH "Practice Guidelines") |
| S4 (MH "Practice Guidelines") OR ( ‘guideline*’ or ‘consensus’ or ‘recommendation*’ or ‘guidance’ or ‘standard’ or ‘statement*’ ) |
| S5 PT practice guidelines |
| S6 S4 OR S5 |
| S7 S2 AND S6 |
| S8 S2 AND S6 Published Date: 20110101-20221231 |
| S9 S2 AND S6 Published Date: 20110101-20221231 english |

S9 Result:664

NICE

‘helicobacter pyloric’ or ‘Campylobacter pyloric’ or ‘h pylori’ or ‘Helicobacter’ or ‘campylobacter’ or ‘pyloric’

Result:0

AHRQ

helicobacter pyloric

Result:3

Campylobacter pyloric

Result:1

SIGN

helicobacter pyloric

Result:0

Campylobacter pyloric

Result:0

GIN

helicobacter pyloric

Result:0

Campylobacter pyloric

Result:0

NHMRC

Search Terms: title_search:(Campylobacter pyloric) OR title_search:(helicobacter pyloric)

Time Period (from):[2011](https://researchdata.edu.au/search/)

Time Period (to):2022

Result:0

WHO

helicobacter pyloric

Result:0

Campylobacter pyloric

Result:0
