# Supplementary material for: Quality appraisal of clinical guidelines for Helicobacter pylori infection and systematic analysis of the level of evidence for recommendations
Source: PLoS One. 2024 Apr 10;19(4):e0301006. doi: 10.1371/journal.pone.0301006 (PMC11006150; doi:10.1371/journal.pone.0301006)
Supplement: S2 Table — (DOCX) [file pone.0301006.s004.docx]

**Supplementary Table 2.** [Descriptive statistic](javascript:;)s of characteristics of included CPGs.

| Characteristic | n (%) |
| --- | --- |
| Type of development organization |  |
| Medical society | 14 (58.3%) |
| Expert panel | 8 (33.3%) |
| Government | 2 (8.3%) |
| Country |  |
| Developed country | 16 (66.7%) |
| Developing country | 8 (33.3%) |
| Version |  |
| Updated | 14 (58.3%) |
| First | 10 (41.7%) |
| Development method |  |
| EB | 18 (75.0%) |
| CB | 6 (25.0%) |
| Used CPG quality tool |  |
| Yes | 15 (62.5%) |
| No | 7 (29.2%) |
| Not stated | 2 (8.3%) |
| Included CPG methodologist |  |
| No | 12 (50.0%) |
| Not stated | 9 (37.5%) |
| Yes | 3 (12.5%) |
| Used grading system |  |
| Yes | 18 (75.0%) |
| No | 4 (16.7%) |
| Not stated | 2 (8.3%) |
| Funding sources |  |
| Yes | 13 (54.2%) |
| No | 7 (29.2%) |
| Not stated | 4 (16.7%) |
| Scope |  |
| Treatment | 8 (33.3%) |
| Diagnosis, treatment | 13 (54.2%) |
| Diagnosis, treatment, prevention | 3 (12.5%) |
| Stated conflict of interest |  |
| Yes | 11 (45.8%) |
| No | 9 (37.5%) |
| Not stated | 4 (16.7%) |
| Year |  |
| ≤2016 | 8 (33.3%) |
| >2016 | 16 (66.7%) |

CPG, clinical practice guideline; EB, evidence-based; CB, consensus-based.
