# Supplementary material for: Quality appraisal of clinical guidelines for Helicobacter pylori infection and systematic analysis of the level of evidence for recommendations
Source: PLoS One. 2024 Apr 10;19(4):e0301006. doi: 10.1371/journal.pone.0301006 (PMC11006150; doi:10.1371/journal.pone.0301006)
Supplement: S3 Table — (DOCX) [file pone.0301006.s005.docx]

**Supplementary Table 3.** Inter-rater reliability for AGREE Ⅱ domain and overall rating.

| AGREE Ⅱ domain and overall rating | ICC (95%CI) |
| --- | --- |
| Domain 1: scope and purpose | 0.840 (0.775, 0.891) |
| Domain 2: stakeholder involvement | 0.935 (0.905, 0.956) |
| Domain 3: rigour of development | 0.927 (0.908, 0.942) |
| Domain 4: clarity of presentation | 0.757 (0.667, 0.831) |
| Domain 5: applicability | 0.812 (0.749, 0.864) |
| Domain 6: editorial independence | 0.923 (0.879, 0.953) |
| Overall rating | 0.801 (0.652, 0.900) |

AGREE: Appraisal of Guidelines for Research and Evaluation; ICC: intraclass correlation coefficients; CI: confidence interval.
