# Supplementary material for: Quality appraisal of clinical guidelines for Helicobacter pylori infection and systematic analysis of the level of evidence for recommendations
Source: PLoS One. 2024 Apr 10;19(4):e0301006. doi: 10.1371/journal.pone.0301006 (PMC11006150; doi:10.1371/journal.pone.0301006)
Supplement: S5 Table — (DOCX) [file pone.0301006.s007.docx]

**Supplementary Table 5.** Overall mean (SD) scores for each AGREE II item of included CPGs.

| AGREE II item | Mean ± SD |
| --- | --- |
| 1 | 6.3 ± 1.1 |
| 2 | 5.5 ± 1.2 |
| 3 | 4.6 ± 1.7 |
| 4 | 4.8 ± 1.8 |
| 5 | 2.0 ± 1.7 |
| 6 | 4.2 ± 1.9 |
| 7 | 3.4 ± 2.4 |
| 8 | 2.4 ± 2.0 |
| 9 | 4.9 ± 1.4 |
| 10 | 4.8 ± 2.2 |
| 11 | 4.7 ± 1.3 |
| 12 | 5.0 ± 1.3 |
| 13 | 2.3 ± 2.0 |
| 14 | 1.4 ± 0.8 |
| 15 | 5.7 ± 1.0 |
| 16 | 5.5 ± 1.0 |
| 17 | 5.1 ± 1.4 |
| 18 | 2.4 ± 1.2 |
| 19 | 1.0 ± 0.0 |
| 20 | 2.7 ± 1.2 |
| 21 | 3.8 ± 0.9 |
| 22 | 2.2 ± 1.8 |
| 23 | 3.1 ± 2.0 |

CPG, clinical practice guideline; AGREE, Appraisal of Guidelines for Research and Evaluation.
