# Supplementary material for: Quality appraisal of clinical guidelines for Helicobacter pylori infection and systematic analysis of the level of evidence for recommendations
Source: PLoS One. 2024 Apr 10;19(4):e0301006. doi: 10.1371/journal.pone.0301006 (PMC11006150; doi:10.1371/journal.pone.0301006)
Supplement: S7 Table — (DOCX) [file pone.0301006.s009.docx]

**Supplementary Table 7.** Overall mean (SD) scores for each AGREE-REX item of included CPGs.

| AGREE-REX item | Mean ± SD |
| --- | --- |
| 1 | 4.5 ± 1.2 |
| 2 | 3.9 ± 1.0 |
| 3 | 4.4 ± 1.4 |
| 4 | 1.6 ± 0.8 |
| 5 | 2.0 ± 1.5 |
| 6 | 1.8 ± 1.3 |
| 7 | 2.6 ± 1.3 |
| 8 | 4.3 ± 1.3 |
| 9 | 3.2 ± 1.4 |

CPG, clinical practice guideline; AGREE-REX: Appraisal of Guidelines for Research and Evaluation-Recommendation Excellence.
