# Supplementary material for: Quality appraisal of clinical guidelines for Helicobacter pylori infection and systematic analysis of the level of evidence for recommendations
Source: PLoS One. 2024 Apr 10;19(4):e0301006. doi: 10.1371/journal.pone.0301006 (PMC11006150; doi:10.1371/journal.pone.0301006)
Supplement: S8 Table — (DOCX) [file pone.0301006.s010.docx]

**Supplementary Table 8.** RIGHT domain scores of included CPGs.

| CPG ID | Basic information | Background | Evidence | Recommendations | Review and quality assurance | Funding and declaration and management of  interests | Other information |
| --- | --- | --- | --- | --- | --- | --- | --- |
| DSG [1] | 50 | 38 | 0 | 14 | 0 | 0 | 0 |
| WGO [2] | 50 | 44 | 0 | 57 | 0 | 0 | 33 |
| KCHUGR [3] | 100 | 75 | 80 | 64 | 100 | 63 | 100 |
| Italian Expert Group [4] | 100 | 56 | 80 | 71 | 50 | 0 | 0 |
| DGVS [5] | 67 | 88 | 80 | 71 | 50 | 75 | 0 |
| ACG [6] | 67 | 69 | 80 | 43 | 0 | 50 | 83 |
| ESPGHAN & NASPGHAN [7] | 50 | 75 | 80 | 50 | 0 | 50 | 33 |
| JSHR [8] | 67 | 81 | 80 | 71 | 0 | 50 | 0 |
| JSPGHAN [9] | 50 | 38 | 0 | 14 | 0 | 0 | 0 |
| KCHUGR [10] | 100 | 63 | 100 | 71 | 100 | 25 | 67 |
| SIGE & SIED [11] | 33 | 75 | 80 | 57 | 50 | 25 | 0 |
| Egyptian Expert Group [12] | 50 | 56 | 20 | 50 | 0 | 0 | 50 |
| NBEHPM [13] | 50 | 44 | 40 | 64 | 0 | 75 | 17 |
| U.S. Expert Group [14] | 33 | 56 | 60 | 71 | 100 | 0 | 67 |
| DGMUHC [15] | 50 | 56 | 80 | 71 | 0 | 25 | 100 |
| HSG [16] | 50 | 56 | 60 | 86 | 0 | 50 | 0 |
| CMS & CSG & HPPUSG [17] | 67 | 56 | 60 | 100 | 0 | 25 | 0 |
| International Expert Group [18] | 50 | 75 | 60 | 86 | 50 | 25 | 33 |
| CSG [19] | 50 | 63 | 70 | 71 | 50 | 25 | 67 |
| IHPWG [20] | 67 | 50 | 60 | 79 | 0 | 50 | 33 |
| CAHPS [21] | 83 | 63 | 60 | 71 | 0 | 50 | 100 |
| GPS [22] | 17 | 50 | 0 | 14 | 0 | 50 | 0 |
| IHPSG [23] | 67 | 50 | 0 | 14 | 0 | 0 | 0 |
| MGA [24] | 33 | 63 | 60 | 79 | 0 | 50 | 0 |
| Mean ± SD | 58.4 ± 21.5 | 60.0 ± 13.4 | 53.8 ± 32.3 | 60.0 ± 24.4 | 22.9 ± 36.1 | 31.8 ± 25.3 | 32.6 ± 37.3 |

CPG, clinical practice guideline; WGO, World Gastroenterology Organization; DGVS, Deutsche Gesellschaft für Gastroenterologie, Verdauungsund Stoffwechselkrankheiten; ACG, American College of Gastroenterology; JSPGHAN, the Japanese Society for Pediatric Gastroenterology, Hepatology and Nutrition; SIGE, the Italian society of gastroenterology; SIED, the Italian society of digestive endoscopy; NBEHPM, Brazilian Nucleus for the Study of Helicobacter pylori and Microbiota; IHPWG, Irish Helicobacter pylori Working Group; USA, the United States of America; KCHUGR, Korean College of Helicobacter and Upper Gastrointestinal Research; JSHR, The Japanese Society for Helicobacter Research; KCHUGR, Korean College of Helicobacter and Upper Gastrointestinal R; DGMUHC, Division of Gastroenterology, McGill University Health Centre; HSG, Hellenic Society of Gastroenterology; CSG, Chile Society of Gastroenterology; CAHPS, Chinese Alliance for Helicobacter pylori Study; IHPSG, Indonesian Helicobacter pylori Study Group; MGA, Mexican Gastroenterological Association; DSG, Danish Society for Gastroenterology; GPS, Gastrointestinal Pathology Society; CMS & CSG & HPPUSG, the Chinese Medical Association & Chinese Society of Gastroenterology & H. pylori and peptic ulcer study group; ESPGHAN & NASPGHAN, European Society for Paediatric Gastroenterology Hepatology and Nutrition & North American Society for Pediatric Gastroenterology, Hepatology and Nutrition; RIGHT: Reporting Items for Practice Guidelines in Healthcare.

**References:**

1. Bytzer PEA (2011) Diagnosis and treatment of Helicobacter pylori in- fection. Dan Med Bull 58: C4271.

2. Hunt RH, Xiao SD, Megraud F, Leon-Barua R, Bazzoli F, et al. (2011) Helicobacter pylori in developing countries. World Gastroenterology Organisation Global Guideline. J Gastrointestin Liver Dis 20: 299-304.

3. Kim SG, Jung HK, Lee HL, Jang JY, Lee H, et al. (2014) Guidelines for the diagnosis and treatment of Helicobacter pylori infection in Korea, 2013 revised edition. J Gastroenterol Hepatol 29: 1371-1386.

4. Zagari RM, Romano M, Ojetti V, Stockbrugger R, Gullini S, et al. (2015) Guidelines for the management of Helicobacter pylori infection in Italy: The III Working Group Consensus Report 2015. Dig Liver Dis 47: 903-912.

5. Fischbach W, Malfertheiner P, Lynen JP, Bolten W, Bornschein J, et al. (2016) [S2k-guideline Helicobacter pylori and gastroduodenal ulcer disease]. Z Gastroenterol 54: 327-363.

6. Chey WD, Leontiadis GI, Howden CW, Moss SF (2017) ACG Clinical Guideline: Treatment of Helicobacter pylori Infection. Am J Gastroenterol 112: 212-239.

7. Jones NL, Koletzko S, Goodman K, Bontems P, Cadranel S, et al. (2017) Joint ESPGHAN/NASPGHAN Guidelines for the Management of Helicobacter pylori in Children and Adolescents (Update 2016). J Pediatr Gastroenterol Nutr 64: 991-1003.

8. Kato M, Ota H, Okuda M, Kikuchi S, Satoh K, et al. (2019) Guidelines for the management of Helicobacter pylori infection in Japan: 2016 Revised Edition. Helicobacter 24: e12597.

9. Kato S, Shimizu T, Toyoda S, Gold BD, Ida S, et al. (2020) The updated JSPGHAN guidelines for the management of Helicobacter pylori infection in childhood. Pediatr Int 62: 1315-1331.

10. Jung HK, Kang SJ, Lee YC, Yang HJ, Park SY, et al. (2021) Evidence-Based Guidelines for the Treatment of Helicobacter pylori Infection in Korea 2020. Gut Liver 15: 168-195.

11. Romano M, Gravina AG, Eusebi LH, Pellegrino R, Palladino G, et al. (2022) Management of Helicobacter pylori infection: Guidelines of the Italian Society of Gastroenterology (SIGE) and the Italian Society of Digestive Endoscopy (SIED). Dig Liver Dis 54: 1153-1161.

12. Alboraie M, Elhossary W, Aly OA, Abbas B, Abdelsalam L, et al. (2019) Egyptian recommendations for management of Helicobacter pylori infection: 2018 report. Arab J Gastroenterol 20: 175-179.

13. Coelho L, Marinho JR, Genta R, Ribeiro LT, Passos M, et al. (2018) IVTH BRAZILIAN CONSENSUS CONFERENCE ON HELICOBACTER PYLORI INFECTION. Arq Gastroenterol 55: 97-121.

14. El-Serag HB, Kao JY, Kanwal F, Gilger M, LoVecchio F, et al. (2018) Houston Consensus Conference on Testing for Helicobacter pylori Infection in the United States. Clin Gastroenterol Hepatol 16: 992-1002.

15. Fallone CA, Chiba N, van Zanten SV, Fischbach L, Gisbert JP, et al. (2016) The Toronto Consensus for the Treatment of Helicobacter pylori Infection in Adults. Gastroenterology 151: 51-69.

16. Georgopoulos SD, Michopoulos S, Rokkas T, Apostolopoulos P, Giamarellos E, et al. (2020) Hellenic consensus on Helicobacter pylori infection. Ann Gastroenterol 33: 105-124.

17. Liu WZ, Xie Y, Lu H, Cheng H, Zeng ZR, et al. (2018) Fifth Chinese National Consensus Report on the management of Helicobacter pylori infection. Helicobacter 23: e12475.

18. Malfertheiner P, Megraud F, O'Morain CA, Gisbert JP, Kuipers EJ, et al. (2017) Management of Helicobacter pylori infection-the Maastricht V/Florence Consensus Report. Gut 66: 6-30.

19. Rollan A, Arab JP, Camargo MC, Candia R, Harris P, et al. (2014) Management of Helicobacter pylori infection in Latin America: a Delphi technique-based consensus. World J Gastroenterol 20: 10969-10983.

20. Smith S, Boyle B, Brennan D, Buckley M, Crotty P, et al. (2017) The Irish Helicobacter pylori Working Group consensus for the diagnosis and treatment of H. pylori infection in adult patients in Ireland. Eur J Gastroenterol Hepatol 29: 552-559.

21. Ding SZ, Du YQ, Lu H, Wang WH, Cheng H, et al. (2022) Chinese Consensus Report on Family-Based Helicobacter pylori Infection Control and Management (2021 Edition). Gut 71: 238-253.

22. Batts KP, Ketover S, Kakar S, Krasinskas AM, Mitchell KA, et al. (2013) Appropriate use of special stains for identifying Helicobacter pylori: Recommendations from the Rodger C. Haggitt Gastrointestinal Pathology Society. Am J Surg Pathol 37: e12-e22.

23. Syam AF, Simadibrata M, Makmun D, Abdullah M, Fauzi A, et al. (2017) National Consensus on Management of Dyspepsia and Helicobacter pylori Infection. Acta Med Indones 49: 279-287.

24. Bosques-Padilla FJ, Remes-Troche JM, González-Huezo MS, Pérez-Pérez G, Torres-López J, et al. (2018) The fourth Mexican consensus on Helicobacter pylori. Rev Gastroenterol Mex (Engl Ed) 83: 325-341.
