# Supplementary material for: Quality appraisal of clinical guidelines for Helicobacter pylori infection and systematic analysis of the level of evidence for recommendations
Source: PLoS One. 2024 Apr 10;19(4):e0301006. doi: 10.1371/journal.pone.0301006 (PMC11006150; doi:10.1371/journal.pone.0301006)
Supplement: S9 Table — (DOCX) [file pone.0301006.s011.docx]

**Supplementary Table 9.** Overall mean (SD) scores for each RIGHT item of included CPGs.

| RIGHT item | Mean ± SD |
| --- | --- |
| 1a | 0.8 ± 0.4 |
| 1b | 0.4 ± 0.5 |
| 1c | 0.6 ± 0.5 |
| 2 | 0.6 ± 0.5 |
| 3 | 0.3 ± 0.5 |
| 4 | 0.7 ± 0.5 |
| 5 | 0.9 ± 0.3 |
| 6 | 0.9 ± 0.2 |
| 7a | 0.8 ± 0.4 |
| 7b | 0.3 ± 0.4 |
| 8a | 0.5 ± 0.5 |
| 8b | 0.0 ± 0.2 |
| 9a | 0.8 ± 0.4 |
| 9b | 0.5 ± 0.4 |
| 10a | 0.7 ± 0.5 |
| 10b | 0.1 ± 0.2 |
| 11a | 0.7 ± 0.5 |
| 11b | 0.4 ± 0.5 |
| 12 | 0.8 ± 0.5 |
| 13a | 1.0 ± 0.0 |
| 13b | 0.7 ± 0.5 |
| 13c | 0.8 ± 0.4 |
| 14a | 0.1 ± 0.3 |
| 14b | 0.4 ± 0.4 |
| 14c | 0.5 ± 0.5 |
| 15 | 0.8 ± 0.4 |
| 16 | 0.3 ± 0.5 |
| 17 | 0.2 ± 0.4 |
| 18a | 0.5 ± 0.5 |
| 18b | 0.1 ± 0.3 |
| 19a | 0.6 ± 0.5 |
| 19b | 0.0 ± 0.2 |
| 20 | 0.4 ± 0.5 |
| 21 | 0.3 ± 0.5 |
| 22 | 0.3 ± 0.4 |

CPG, clinical practice guideline; RIGHT: Reporting Items for Practice Guidelines in Healthcare.
