# Supplementary material for: Quality appraisal of clinical guidelines for Helicobacter pylori infection and systematic analysis of the level of evidence for recommendations
Source: PLoS One. 2024 Apr 10;19(4):e0301006. doi: 10.1371/journal.pone.0301006 (PMC11006150; doi:10.1371/journal.pone.0301006)
Supplement: S12 Table — (DOCX) [file pone.0301006.s014.docx]

**Supplementary Table 12.** Overall mean (SD) and median (Q1–Q3) of the number of level of evidence and strength of recommendation.

|  | **Mean (SD) Median (Q1-Q3)** |
| --- | --- |
| High | 9.6 (6.3) 8.5 (5.0-13.8) |
| Moderate | 7.5 (6.4) 5.0 (5.0-9.8) |
| Low | 6.2 (4.5) 5.0 (4.0-8.5) |
| Very low | 4.8 (6.3) 3.0 (0.2-5.8) |
| Strong | 18.0 (9.8) 15.5 (13.0-20.2) |
| Weak | 10.1 (7.3) 9.5 (4.2-16.0) |
