# Supplementary material for: Quality appraisal of clinical guidelines for Helicobacter pylori infection and systematic analysis of the level of evidence for recommendations
Source: PLoS One. 2024 Apr 10;19(4):e0301006. doi: 10.1371/journal.pone.0301006 (PMC11006150; doi:10.1371/journal.pone.0301006)
Supplement: S13 Table — (DOCX) [file pone.0301006.s015.docx]

**Supplementary Table 13.** The number of level of evidence and strength of recommendation for different subgroups of evidence-based CPGs (Mean ± SD, %).

| **Subgroups** | **Statistics** | **High** | **Moderate** | **Low** | **Very low** | **Strong** | **Weak** |
| --- | --- | --- | --- | --- | --- | --- | --- |
| Type of development organization |  | P = 0.174 | P = 0.260 | P = 0.272 | P = 0.678 | P = 0.396 | P = 0.457 |
| Medical society | 10 (55.6%) | 8.7 ± 5.7 | 9.7 ± 7.4 | 7.6 ± 5.3 | 4.0 ± 5.5 | 19.7 ± 11.6 | 10.3 ± 6.6 |
| Expert panel | 6 (33.3%) | 13.0 ± 7.0 | 5.2 ± 3.8 | 5.0 ± 2.8 | 6.7 ± 8.5 | 18.2 ± 6.9 | 11.7 ± 9.1 |
| Government | 2 (11.1%) | 4.0 ± 0.0 | 3.5 ± 2.1 | 2.5 ± 0.7 | 3.0 ± 0.0 | 9.0 ± 4.2 | 4.0 ± 1.4 |
| Country |  | P = 0.666 | P = 0.707 | P = 0.916 | P = 0.960 | P = 0.846 | P = 0.913 |
| Developed country | 12 (66.7%) | 10.1 ± 7.4 | 7.1 ± 6.2 | 6.2 ± 4.0 | 4.8 ± 6.5 | 18.3 ± 10.0 | 9.9 ± 7.5 |
| Developing country | 6 (33.3%) | 8.7 ± 3.8 | 8.3 ± 7.2 | 6.0 ± 5.9 | 4.7 ± 6.4 | 17.3 ± 10.5 | 10.3 ± 7.6 |
| Version |  | P = 0.417 | P = 0.449 | P = 0.176 | P = 0.681 | P = 0.299 | P = 0.664 |
| Updated | 13 (72.2%) | 10.4 ± 6.6 | 8.2 ± 7.3 | 7.1 ± 4.9 | 4.4 ± 6.1 | 19.5 ± 10.9 | 10.5 ± 7.0 |
| First | 5 (27.8%) | 7.6 ± 5.7 | 5.6 ± 2.6 | 3.8 ± 2.2 | 5.8 ± 7.4 | 14.0 ± 5.0 | 8.8 ± 8.7 |
| Used CPG quality tool |  | P = 0.322 | P = 0.415 | P = 0.947 | P = 0.003 | P = 1.000 | P = 0.008 |
| Yes | 15 (83.3%) | 8.9 ± 5.7 | 8.1 ± 6.7 | 6.1 ± 4.7 | 3.0 ± 4.6 | 18.0 ± 10.1 | 8.1 ± 6.3 |
| No | 3 (16.7%) | 13.0 ± 9.5 | 4.7 ± 3.5 | 6.3 ± 4.0 | 13.7 ± 6.7 | 18.0 ± 10.1 | 19.7 ± 3.2 |
| Included CPG methodologist |  | P = 0.452 | P = 0.426 | P = 0.670 | P = 0.212 | P = 0.470 | P = 0.202 |
| No | 10 (55.6%) | 9.5 ± 6.4 | 7.2 ± 5.6 | 6.8 ± 4.8 | 7.1 ± 7.7 | 18.6 ± 9.3 | 12.0 ± 7.9 |
| Not stated | 5 (27.8%) | 12.0 ± 7.5 | 10.2 ± 9.1 | 6.2 ± 5.4 | 2.4 ± 1.3 | 20.6 ± 12.8 | 10.2 ± 6.3 |
| Yes | 3 (16.7%) | 6.0 ± 2.6 | 4.0 ± 1.7 | 4.0 ± 1.7 | 1.0 ± 1.7 | 11.7 ± 4.9 | 3.3 ± 2.1 |
| Funding sources |  | P = 0.098 | P = 0.297 | P = 0.425 | P = 0.958 | P = 0.083 | P = 0.834 |
| Yes | 11 (61.1%) | 8.7 ± 6.1 | 5.8 ± 3.3 | 5.9 ± 4.3 | 5.0 ± 6.6 | 14.6 ± 5.8 | 10.8 ± 7.9 |
| No | 5 (27.8%) | 8.0 ± 5.6 | 9.0 ± 6.9 | 8.0 ± 5.4 | 4.8 ± 7.7 | 20.4 ± 11.5 | 9.4 ± 7.9 |
| Not stated | 2 (11.1%) | 18.5 ± 2.1 | 13.0 ± 17.0 | 3.0 ± 2.8 | 3.5 ± 0.7 | 30.5 ± 17.7 | 7.5 ± 3.5 |
| Scope |  | P = 0.741 | P = 0.042 | P = 0.239 | P = 0.613 | P = 0.172 | P = 0.620 |
| Treatment | 8 (44.4%) | 9.4 ± 7.3 | 7.1 ± 6.3 | 8.0 ± 6.0 | 4.1 ± 6.0 | 18.8 ± 10.1 | 9.9 ± 7.6 |
| Diagnosis, treatment | 8 (44.4%) | 9.0 ± 5.0 | 5.4 ± 2.9 | 5.2 ± 1.9 | 6.2 ± 7.3 | 14.5 ± 5.3 | 11.4 ± 7.5 |
| Diagnosis, treatment, prevention | 2 (11.1%) | 13.0 ± 9.9 | 17.5 ± 10.6 | 2.5 ± 3.5 | 1.5 ± 2.1 | 29.0 ± 19.8 | 5.5 ± 6.4 |
| Year |  | P = 0.293 | P = 0.906 | P = 0.265 | P = 0.377 | P = 0.504 | P = 0.171 |
| ≤2016 | 5 (27.8%) | 12.2 ± 6.1 | 7.8 ± 9.8 | 4.2 ± 1.8 | 2.6 ± 2.6 | 20.6 ± 12.7 | 6.2 ± 4.2 |
| >2016 | 13 (72.2%) | 8.6 ± 6.3 | 7.4 ± 5.1 | 6.9 ± 5.1 | 5.6 ± 7.1 | 17.0 ± 8.9 | 11.5 ± 7.8 |

CPG, clinical practice guideline; EB, evidence-based; CB, consensus-based.
